# The unprecedented membrane deformation of the human nuclear envelope, in a magnetic field, indicates formation of nuclear membrane invaginations

**DOI:** 10.1038/s41598-020-61746-0

**Published:** 2020-03-20

**Authors:** Régine Dazzoni, Axelle Grélard, Estelle Morvan, Anthony Bouter, Christopher J. Applebee, Antoine Loquet, Banafshé Larijani, Erick J. Dufourc

**Affiliations:** 1 Institute of Chemistry & Biology of Membranes & Nanoobjects, UMR5248, CNRS, Université Bordeaux, INP-Bordeaux, F-33600 Pessac, France; 2 Cell Biophysics Laboratory, Ikerbasque Basque Foundation for Science, Instituto Biofísika (CSIC, UPV/EHU) and Research Centre for Experimental Marine Biology and Biotechnology (PiE), University of the Basque Country (UPV/EHU), Leioa, Spain; 3 Institut Européen de Chimie et Biologie, UMS3033, CNRS, Université Bordeaux, INSERM (US001), 2 rue Escarpit, Pessac, 33600 France; 4 Cell Biophysics Laboratory, Centre for Therapeutic Innovation & Department of Pharmacy and Pharmacology, & Department of Physics, University of Bath, Bath, United Kingdom

**Keywords:** Biophysics, Membrane biophysics, Membrane structure and assembly

## Abstract

Human nuclear membrane (hNM) invaginations are thought to be crucial in fusion, fission and remodeling of cells and present in many human diseases. There is however little knowledge, if any, about their lipid composition and dynamics. We therefore isolated nuclear envelope lipids from human kidney cells, analyzed their composition and determined the membrane dynamics after resuspension in buffer. The hNM lipid extract was composed of a complex mixture of phospholipids, with high amounts of phosphatidylcholines, phosphatidylinositols (PI) and cholesterol. hNM dynamics was determined by solid-state NMR and revealed that the lamellar gel-to-fluid phase transition occurs below 0 °C, reflecting the presence of elevated amounts of unsaturated fatty acid chains. Fluidity was higher than the plasma membrane, illustrating the dual action of Cholesterol (ordering) and PI lipids (disordering). The most striking result was the large magnetic field-induced membrane deformation allowing to determine the membrane bending elasticity, a property related to hydrodynamics of cells and organelles. Human Nuclear Lipid Membranes were at least two orders of magnitude more elastic than the classical plasma membrane suggesting a physical explanation for the formation of nuclear membrane invaginations.

## Introduction

Cell nuclei are known to have a quasi-spherical shape at rest with a diameter that may vary from 5 to 10 µm depending on the cell type. Their envelope is composed of a double lipid bilayer, the inner and outer nuclear membranes (NM), and the nuclear lamina. The lamina play an important role in structure, stability, and gene regulation and consist of a dense network of proteins underlaying the inner membrane^1^. Inner and outer NM join at the nuclear pores and enclose a perinuclear space that is continuous with the endoplasmic reticulum (ER), providing a reservoir of membrane material that may be used during nuclear membrane formation. Powerful imaging techniques highlighted the presence of nuclear envelope substructures forming invaginations inside the nucleus^2^. These invaginations have been called Nucleoplasmic Reticula (NR). NRs are reported in numerous normal and abnormal cells from the plant and animal kingdom. NRs are particularly abundant in many tumor cell types including brain, breast, kidney, bladder, prostate and ovary and suggest that the NRs regulatory mechanisms are susceptible to pathological dysregulation^3,4^. However, and to the best of our knowledge, their role is still unknown. The appearance of this specific morphology in the nucleus suggests that NRs may have a role in mechano-transduction of signal to the nucleoplasm, gene expression, RNA trafficking, and cell differentiation^1,5^. NRs are stable and persistent but are also flexible enough to accommodate nuclear rotation or changes in morphology^6^. In live cells, NR may change on a timescale of minutes^2^. The NR regulation appears to be a dynamic process that may be controlled by several pathways. NR formation might be controlled by changes in phospholipid bilayer composition when choline phosphate cytidylyl-transferase (CCTα) is activated. This enzyme synthesizes phosphatidylcholine (PC), which might play a role in modifying the human nuclear membrane (hNM) mechanical properties and promote the development of invaginations^7^. Unlike the sea urchin model organism hNM dynamics has not been investigated yet. Larijani *et al*.^8^ have shown the conserved membrane fusion mechanism in mammlian cells and sea urchins; both proteins and lipids are essential requirements for such a process^9^. In the sea urchin model, an elevated heterogeneity has been reported for such nuclear membranes and high amounts of cholesterol and polyphosphoinositides were found. Fluid properties were nonetheless reported, offering an alternative to the present paradigm that cholesterol-enriched membranes are rigid membranes^10^. To complement these findings the essential role of diacylglycerol as both a second messenger and a modulator of membrane dynamics in the formation of the nuclear membrane was also demonstrated^9^. However, we could not go into membrane elasticity considerations, as done herein, due to a much lower S/N ratio of our previous results.

**Figure 1:**
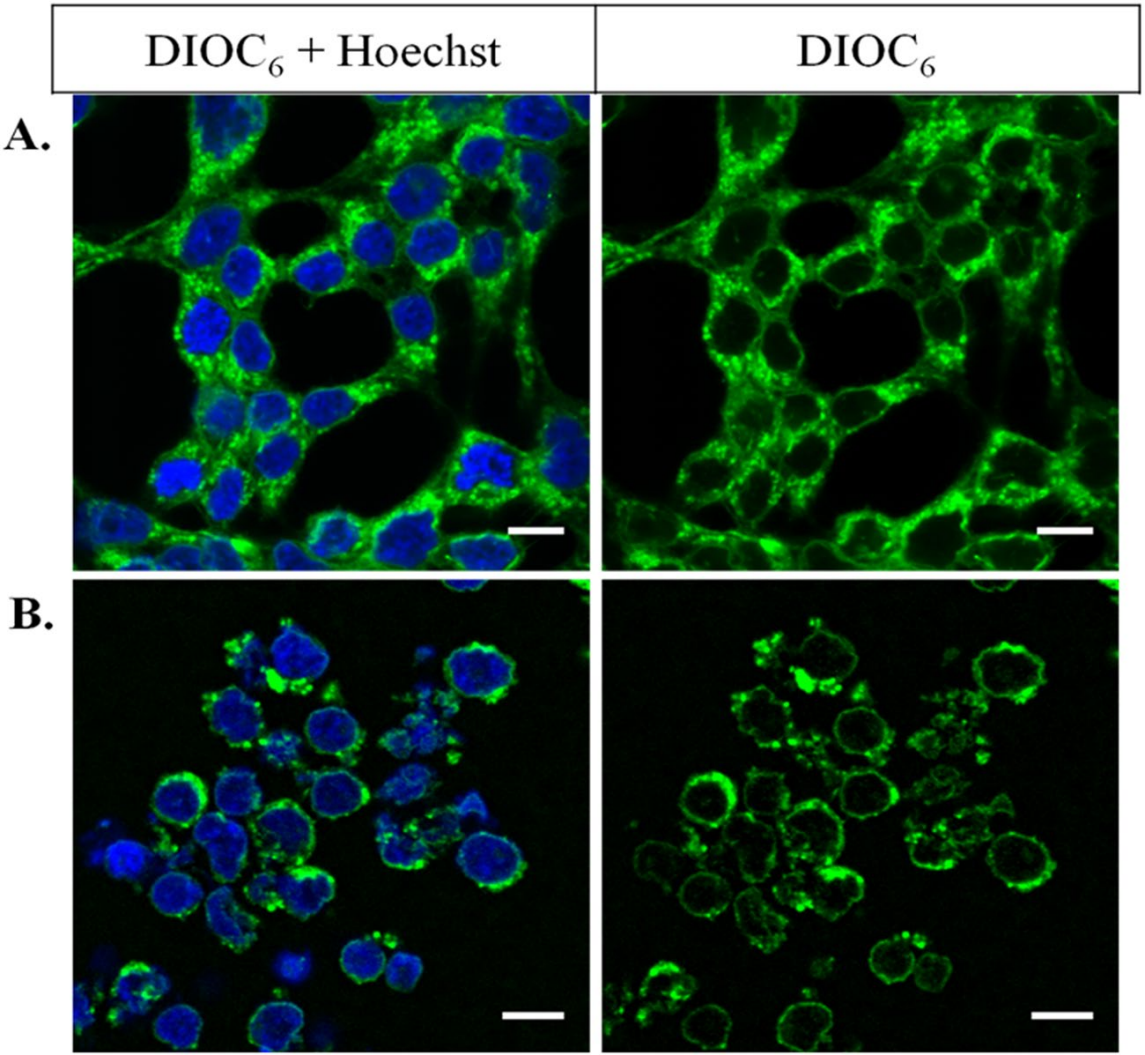
Nuclei purification efficiency by the nitrogen gas cavitation method. HEK 293 T Cells (**A**) and cells treated by the cavitation method (**B**). Staining by DiOC_6_ (1 µM, green) to visualize membranes, and by Hoechst (1 µM, blue), binding specifically to chromatin and imaging the nuclei interior. Images have been acquired by an inverted confocal instrument with a 63-fold water immersion objective. Excitation wavelengths of DiOC_6_ and Hoechst were respectively 488 nm and 405 nm; emission ranges are of 504–561 nm for DiOC_6_ and of 436–483 nm for Hoechst. Scale bars: 10 µm.

To the best of our knowledge, a detailed characterization of the lipid composition and physical properties of the human nuclear membranes by a non-invasive methodology has not been reported. One of the most precise, quantitative and non-invasive methods for studying physical properties of natural membranes, such as composition, dynamics and morphology, is NMR spectroscopy^11–13^. Phosphorous, proton and carbon liquid-state NMR were chosen to probe phospholipid composition, whereas phosphorous and deuterium solid-state NMR were used to monitor the physical properties (fluidity, elasticity) of human nuclear membranes. In order to provide enough material for NMR and determine membrane dynamics and composition without presence of detergent, nuclei needed to be purified using a non-detergent method. In the past, the use of detergents for purifying nuclei from mammalian cells led to misinterpretations of function, localization and composition of the “nuclear lipids”^14,15^. Based on existing procedure^16^ we therefore implemented a non-detergent method for purifying nuclei from Human Embryonic Kidney (HEK) 293 T cells (Fig. 1). Lipids were extracted from quasi-pure nuclei with minimum contamination from the endoplasmic reticulum. The extracted lipids were reconstituted to reform membrane bilayers with their natural, as opposed to synthetic, lipid composition. We thus, consider this approach to be non-invasive. Furthermore NMR experiments do not affect the spatial arrangements of the reconstituted natural membrane bilayers.

Using various types of advanced liquid-state and solid-state NMR experiments we report herein the atypical lipid composition and physical properties of human Nuclear Membrane. The most striking result is the significant deformation of the reconstructed nuclear lipid membrane in magnetic fields. In addition, we show that such a deformation is directly linked to a very high nuclear membrane elasticity, two orders of magnitude more elastic than that of plasma membrane lipids. We discuss the biological implications of this membrane elasticity in terms of membrane invaginations.

## Results

### Purification of large amounts of human Nuclear Membrane lipids using a non-detergent method

The method published by Blober & Potter^16^ was optimized and used to recover mg-scale lipid membranes without detergent. Human Embryonic Kidney (HEK) 293 T cells were grown as described in Materials & Methods. They were visualized using Hoechst and DiOC_6_ dyes that specifically bind to nuclei and membranes as respectively seen in blue and green in Fig. 1A, using fluorescence microscopy. DiOC_6_ staining shows all membranes of HEK cells: the plasma membrane surrounding the cell, the Endoplasmic Reticulum (ER) and the nuclear membrane enclosing the decondensed chromatin stained by the Hoechst reagent. Nuclei were submitted to a nitrogen gas cavitation action provided by a cell disruption bomb apparatus. Figure 1B shows the efficiency of the method: the plasma membrane is absent; nuclei are intact and nuclear membranes appear complete. As the outer nuclear membrane is continuous with the ER, a small proportion of ER membranes is nonetheless observed attached to nuclei. As it is minor no further removal has been performed. Lipids were then extracted from the nuclei as described in the Materials & Methods section. The yield is quite important: from ca. 10^8^ nuclei, 4 mg of human nuclear membrane lipids (dry weight) could be recovered.

### Human Nuclear Membranes are mainly composed of phosphatidylcholines, phosphoinositides and cholesterol

Lipid extracts were solubilized and the solution transferred into a 5 mm diameter NMR tube to be analyzed by liquid-state ^1^H-,^13^C- and ^31^P-NMR. The phosphorus 1D spectrum shows a classical series of isotropic lines that can be assigned to individual phospholipid species (Supplementary Fig. 1, bottom). As NMR spectra were acquired under quantitative conditions, the amount of each phospholipid in the extract was obtained by integrating the surface area under the spectral peaks (see spectral simulations of 1D ^31^P-NMR spectra, Supplementary Fig. 1). Spectral areas were normalized with respect to the internal reference of known concentration. We thus determined the total quantity of phospholipids in the extract, from 107×10^6^ nuclei, 4 mg of phospholipids were extracted. Results are reported in Table 1. Phosphatidylcholines (PC) dominate the lipid extract with *ca*. 60%. Negatively charged lipids were present at *ca*. 25% with more than half of them being phosphoinositides (PI). Phosphatidylethanolamines (PE) were present at 9%. ^31^P-NMR chemical shifts globally agree with literature values obtained from individual species. There was however some variance with negatively charged lipids that were very sensitive to pH^17^. Another source of variance was due to the fact that chemical shifts of lipids in a complex natural mixture may slightly vary from those reported from individual synthetic species (Table 1). The identification of most of the main lipids was nonetheless secured using 2D NMR (^1^H-^31^P correlation, Supplementary Fig. 2). Of interest is also the presence of Cholesterol that has been detected using 2D-NMR (^1^H-^13^C correlation, Supplementary Figs. 1 and 3), and also the presence of numerous unsaturation in the lipid chains (5.3 to 5.4 ppm).

**Table 1:** Quantification of phospholipid species in human nuclear membranes (hNM) by liquid-state NMR.

Phospholipid^a^	^31^P-chemical shift (ppm) from literature	^31^P-chemical shift hNM (ppm)^c^	% mol^d^
TPP (reference)		−17.95	
PC	−0.84 ± 0.01	−0.84 ± 0.01	**46 ± 4**
EPC	−0.78 ± 0.01	−0.77 ± 0.01	**15 ± 2**
LPC^e^	−0.28 ± 0.04	−0.22 ± 0.01	0 ± 1
PI	−0.36 ± 0.03	−0.20 ± 0.06	**11 ± 1**
SM	−0.07 ± 0.03	−0.10 ± 0.01	3 ± 2
PS^g^	−0.05 ± 0.01	−0.06 ± 0.01	4 ± 1
PE	0.04 ± 0.10	0.03 ± 0.01	9 ± 2
LPI^e^	0.10 ± 0.04	0.03 ± 0.01	4 ± 1
CL^g^	0.18 ± 0.01	0.06 ± 0.01	1 ± 1
PA	0.23 ± 0.04	0.33 ± 0.02	6 ± 1
PG^f^	0.47 ± 0.01	0.57 ± 0.01	0 ± 1

^a^TPP: Triphenylphosphate (internal reference), PC: phosphatidylcholine, EPC: ether phosphatidylcholine, LPC: lysophosphatidylcholine, PI: phosphatidylinositol, LPI: lysophosphatidylinositol SM: sphingomyelin, PS: phosphatidylserine, PE: phosphatidylethanolamine, CL: cardiolipin, PA: phosphatitic acid, PG: phosphatidylglycerol.

^b^Chemical shifts of individual phospholipids in the MeOD/CDCl_3_ (from Kaffarnik *et al*. and Meneses & Glonek^18,19^). Average over the 2 reference values. Reference H_3_PO_4_ (0 ppm).

^c^Chemical shifts of individual phospholipids in the nuclear lipid extract mixture (4 mg of phospholipids solubilized in of 500 µL of MeOD/CDCl_3_ (1:2) + 30 µl of 0.2 M EDTA-D_2_O pH 6). Chemical shifts were referenced with respect to the internal standard TPP: -17.95ppm^18^, assignment was performed with ^1^H-^31^P 2D NMR (supplementary materials). Values in the column represent the average over 3 different sample extractions over a period of one year.

^d^Molar lipid content in the extract as determined from simulated spectra using the DMFIT software (see Materials and Methods and Supplementary Fig. 1). Figures represent the average over 3 NMR sample preparations. Bold stand for the most abundant species.

^e^Only detected once among 3 samples. Assignment by comparison with literature values.

^f^Only detected once among 3 samples.

^g^Assignment by comparison with literature values.

### Nuclear lipid membranes are fluid below 0 °C

**Figure 2:**
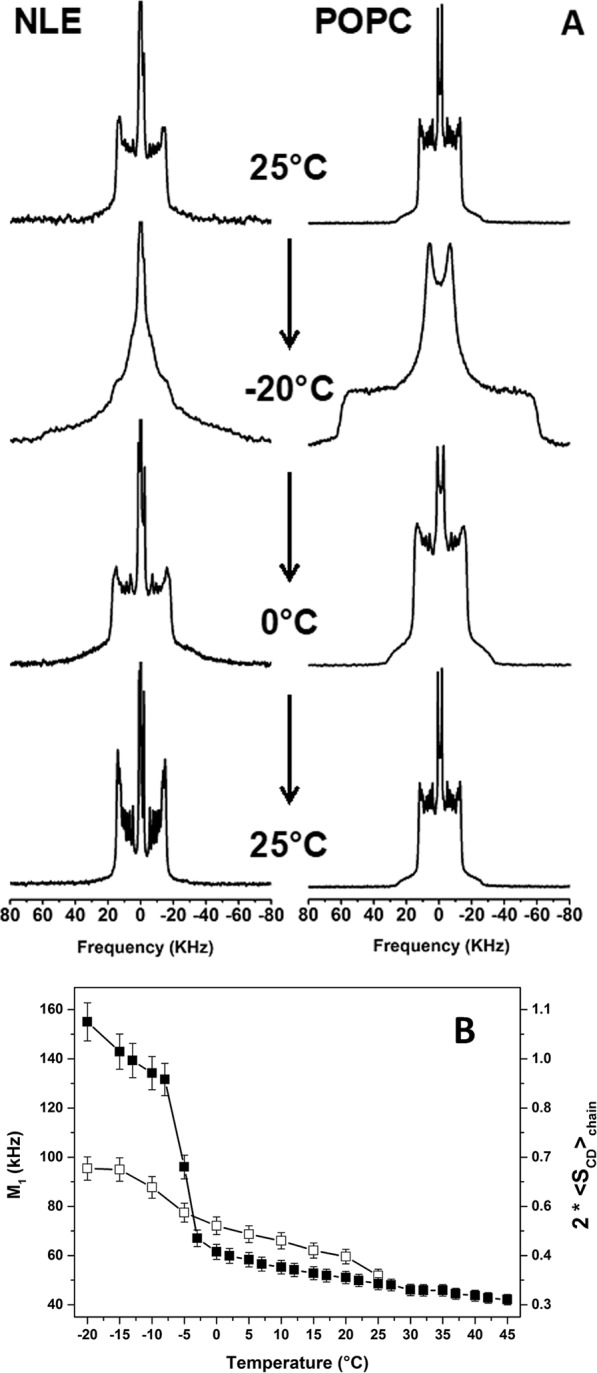
Thermotropism of hNM lipid extract membranes and POPC controls. (**A**) Selected ^2^H-NMR spectra of reconstituted human nuclear lipid membrane extracts (NLE) mixed with ^2^H_31_-POPC and pure ^2^H_31_-POPC (POPC) vesicles during a thermal variation (25 °C down to −20 °C and back) and recorded after temperature stabilization. Spectra were obtained after Fourier transformation of solid-echo type experiments accumulated for 2–18 k transients. The entire thermal variation is shown in Supplementary Fig. 5. Details for experimental parameters and data treatment are in the methods section. **(B)** Thermal variation of the first spectral moment (reporting membrane fluidity), M_1_, from ^2^H-NMR spectra of **A** and Supplementary Fig. 3 of reconstituted NLE (□), and control POPC vesicles (■). The double y-axis plots twice the fatty acid chain order parameter to depict rigid chains (solid-ordered membranes) when 2 *S*_*CD chain*_ = 1 and fully disordered systems (liquid-like) when 2 *S*_*CD chain*_ = 0.

Lipid extracts were mixed with deuterated POPC (10:1 molar ratio) and rehydrated for membrane thermotropic and dynamics studies. Spherical liposomes (multilamellar vesicles, MLV) of 0.9 μm average diameter was readily detected by optical microscopy indicating that the lipid extract was capable of reforming lipid bilayers (Supplementary Fig. 4). The hydrated lipid extract was analyzed between −20 °C to 45 °C by solid-state ^2^H-NMR. As a control, spectra for pure ^2^H_31_-POPC MLV were also recorded. Selected spectra are shown in the Fig. 2A, the spectra for the entire temperature variation are shown in Supplementary Fig. 5. ^2^H_31_-POPC spectra reflect the classical thermotropic behavior of MLVs: uniaxial lamellar fluid phase (also known as, *L*_*α*_, or liquid-disordered, *ld*) above 0 °C with well-resolved quadrupolar doublets, the largest width being ≈ 26 kHz, with marked shoulders at ca. 52 kHz, and below very wide ^2^H-NMR spectra of approximately 120 kHz width, which was typical of C-^2^H_2_ groups no longer undergoing chain and segment isomerization. These were features characteristic of a lamellar gel phase, also known as *L*_*β*_, or solid-ordered phase, *so*,^20^. These features in fluid and gel phases reflect a random distribution of local bilayer normals with respect to the magnetic field, *i*.*e*., “powder” or spherical distribution of bilayer membranes typical of slowly tumbling micrometer-size spherical liposomes^20–22^. This thermal behavior was completely reversible (Fig. 2). Reconstituted nuclear membrane vesicles, labelled with ^2^H_31_-POPC, led to a fluid phase spectrum at 25 °C. The width (≈ 30 kHz) was wider than for pure POPC MLV, indicating that the lipid probe was sensing a more rigid membranous environment. Cooling down to −20 °C led to a composite spectrum with broad features of approximately 120, 35 kHz and an unresolved broad central peak. This indicated a heterogeneous lamellar phase with rigid parts but also with more dynamic portions. Increasing the temperature to 0 °C led to an almost fluid phase spectrum with broad features detected near 110 kHz. Increasing further to 25 °C resulted in a well-resolved spectrum consisting of several doublets. In this case the spectrum lost the “powder” type distribution, *i*.*e*., the “shoulders” at *ca*. 60 kHz were absent. This event suggested a deformation/orientation of the reconstituted membrane vesicles in the magnetic field (*vide infra*).

The temperature behavior of hydrated lipid systems can be further quantified by calculating the first spectral moment, *M*_1_^20,23^,. *M*_1_ is directly proportional to the average chain order parameter, ${S}_{CDchain}=\sqrt{3}{M}_{1}/\pi {A}_{Q}$, with *A*_*Q*_ being the static quadrupolar coupling constant^24^. The chain order parameter is a direct measure of the state of order/disorder of the membrane: 2 *S*_*CD chain*_ = 1 for totally rigid systems and 0 for liquid systems. Figure 2B shows both *M*_1_ and 2 *S*_*CD chain*_. At low temperatures, POPC model membrane reached 2 *S*_*CD chain*_ values near 1 confirming the rigid nature of the hydrophobic membrane core. A sharp order-disorder transition occurs at T_m_ = −5 ± 2 °C due to the onset of various molecular processes^25^. The chain ordering slowly decreased when the temperature was increased above T_m_ also indicative of the liquid disordered state. In contrast, the reconstituted nuclear membrane vesicles did not reach a complete ordered state at the lowest temperature. Their phase transition was broad and occurred between −15 °C to 0 °C. Chain ordering in the fluid phase was greater than that observed with pure POPC vesicles except above 25 °C where both systems show similar fluidity.

### Spectral simulations reveal ordering and vesicle deformability

**Figure 3:**
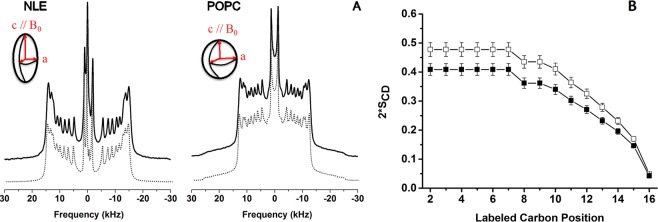
Simulations of deuterium solid state spectra reveals ordering and deformability. **A**) Experimental and simulated (dotted line) ^2^H spectra of reconstructed membrane vesicles and control POPC vesicles as obtained at 25 °C. Same experimental parameters as in Fig. 2. Simulated spectra were calculated as described in Materials & Methods. Inserts (ellipsoid and sphere) depict the deformation that is obtained from simulations, *c/a* = 3.0 for NLE and 1.0 for POPC. *c* and *a* are the long and short semi-axes with *c* being aligned with the B_0_ magnetic field direction. **B)** Plot of *S*_*CD*_ order parameters as a function of the labelled carbon position, *k*, along the palmitoyl acyl chain (*k* = 16, chain end, *k* = 2, membrane interface). Accuracy in order parameters is 0.5%.

For fluid phase temperatures (*e*.*g*., 25 °C) a minute description of chain ordering may be obtained from ^2^H-NMR spectra. Figure 3A shows both experimental and calculated spectra for ^2^H_31_-POPC and reconstituted nuclear membrane vesicles labelled with ^2^H_31_-POPC. The palmitic chain contains 14 methylene (C-^2^H_2_) labelled positions and one C-^2^H_3_. The methyl terminal doublet was assigned as the smallest and the most intense peak^22^. Only 8 other splittings were detected from the experimental spectra indicating that there are several magnetically and dynamically equivalent positions. Simulations allowed to accurately determine splitting values (in kHz) with a high accuracy. Assigning a doublet to a labelled position, *k*, relied on comparisons with values from the literature and using mean-field theories^21^. Figure 3B illustrates the carbon-deuterium order parameter (*S*_*CD*_) profile as a function of the labelled carbon position, *k*, after converting quadrupolar doublets into order parameters ($|\Delta {\nu }_{Q}^{k}|=\frac{3}{4}{A}_{Q}{S}_{CD}^{k}$). A typical behavior was detected with elevated values for positions 2 to 8-10 and a marked decrease towards the chain end, *i*.*e*., the bilayer center. This reflected the well-known gradient of order parameters with increased rigidity near the interface and an almost liquid-like environment at the center of the membrane. The reconstituted nuclear membrane vesicles have a similar ordering profile but are clearly more ordered than pure POPC. Ordering information can be correlated to bilayer thickness by considering that the more motion present in the bilayer core the shortest, in average, are the hydrophobic chains^22,26^. At 25 °C the value for the bilayer thickness of pure POPC was 42.8 Å. 44.2 Å were obtained for reconstituted nuclear membrane vesicles respectively (Supplementary Table 1). The difference is small but notable as the accuracy in determining the hydrocarbon average chain length is ± 0.4 Å. This could be due to the presence of cholesterol that has been shown to increase membrane ordering and hence membrane thickness^23^.

Spectral simulations were performed by considering a distribution of bilayer orientations, *θ*, with respect to the magnetic field as in an ellipsoid of revolution: $p(\theta ) \sim \frac{si{n}^{2}\theta }{si{n}^{2}\theta +\frac{c}{a}co{s}^{2}\theta }$^27^, where *c/a* represents the ellipsoid long axis to short axis ratio^27^. *c/a* values were varied until a correct agreement was obtained between experimental and calculated spectra (Fig. 3A). *c/a* was found near 1.0 for POPC (almost no deformation) and close to 3.0 for reconstituted nuclear membrane vesicles. This indicated a large prolate deformation of the initially spherical vesicles. Inserts in Fig. 3A (ellipsoid and sphere) depict the deformation that was obtained from simulations, with *c* being aligned with the B_0_ magnetic field direction.

### ^31^P-NMR reveals enhanced deformability of NLE vesicles

**Figure 4:**
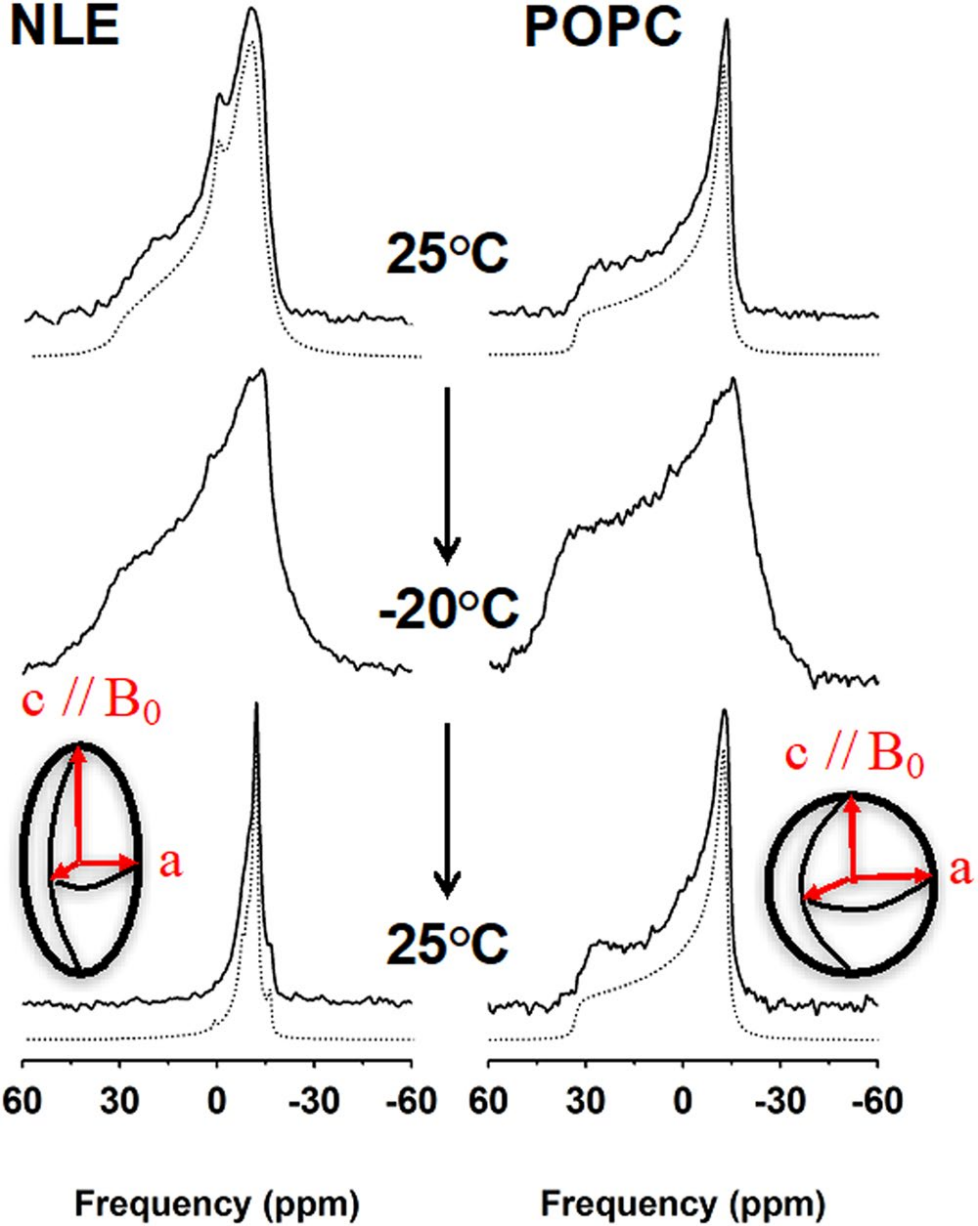
Temperature-dependent ^31^P-NMR spectral lineshapes and simulations of nuclear lipid extract membranes and POPC vesicles. Selected ^31^P-NMR spectra (solid lines) of reconstituted nuclear lipid membrane extracts (NLE) and POPC vesicles during a thermal variation (25 °C down to -20 °C and back) and recorded after temperature stabilization. Spectra were obtained after Fourier transformation of Hahn-echo type experiments accumulated for 300 to 7000 transients. The entire thermal variation is shown in Supplementary Fig. 4. Details for experimental parameters and data treatment are found in the methods section. Simulated spectra (dotted lines) were calculated as described in Materials & Methods. Inserts (ellipsoid and sphere) depict the deformation that is obtained from simulations, *c/a* = 3.0 for NLE and 1.0 for POPC. *c* and *a* are the long and short semi-axes with *c* being aligned with the B_0_ magnetic field direction.

The liposome deformation that was pointed out by deuterium NMR was further investigated by wide line ^31^P-NMR. Selected spectra are shown in Fig. 4 together with POPC control liposomes obtained through the same temperature variations and magnetic conditions (the entire temperature variation is shown in Supplementary Fig. 6). POPC illustrates a classical “powder” spectral profile indicating a random or spherical distribution of bilayer normals in the field (*i*.*e*., non-oriented samples). This line-shape is independently obtained at initial conditions and after having been to low temperatures and back. The reconstituted nuclear membrane vesicles show prominent differences between the initial spectrum taken at 25 °C and the final one recorded after the temperature variations in the magnetic field. The left hand side shoulder almost disappeared and the spectrum looks like an oriented sample spectrum as could be obtained by placing lamellar lipid membranes onto glass plates and orienting the plate normal perpendicular to the field^25^. Such a spectrum may be accounted for by a huge liposome deformation into an ellipsoid prolate with short and long axes, *a* and *c* (Fig. 4 diagrams), such as *c/a* > 1.

This was in complete accordance with what has been observed by deuterium NMR (*vide supra*); the samples were however slightly different; this one was without insertion of the deuterated lipid reporter. As the same deformation values (*c/a*) were found from deuterium and phosphorus NMR on different samples this strengthens the observation of a huge magnetic field effect on lipid nuclear membranes.

#### Magic angle spinning abolishes magnetic field induced vesicle deformation

**Figure 5:**
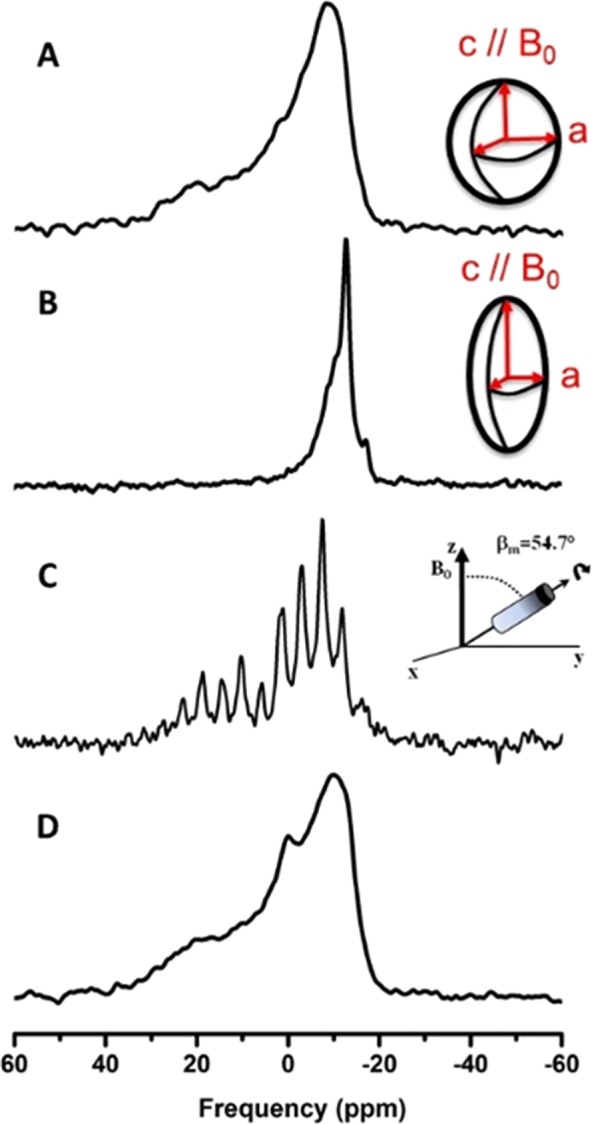
Annihilation of magnetic field-induced vesicle deformation by Magic Angle sample Spinning (MAS) as detected by ^31^P-NMR. (**A**) “Powder” spectrum obtained at 25 °C just after sample preparation and scheme of a sphere indicating a spherical distribution of bilayer normals. (**B)** Oriented-like spectrum at 25 °C, after placing the sample in the magnetic field at low temperatures and returning back to 25 °C. The insert depicts the prolate deformation of vesicles with a *c/a* semi-axes ratio of 3. (**C)** Same as (**B**) except for magic angle sample spinning at 1400 rotor rotations per second (1.4 kHz). Spinning side bands spaced every 1.4 kHz depict the powder pattern line shape, *i*.*e*., loss of deformation. (**D)** Spectrum obtained at 25 °C just after stopping MAS showing the recovery of a powder pattern lineshape similar to (**A)**. All spectra were obtained with the same acquisition parameters (as in Fig. 4).

The magnetic field induced liposomes deformation in general occurs when the magnetic energy for orienting molecules in the field overcomes the curvature elastic energy of liposomes^28^ (Supplementary Information). These two energies can be counterbalanced using a third one, the mechanical energy related to rapid rotation of the sample at the magic angle (54.7°), as it is often performed in solid state NMR. In the present experiment, the sample, once the initial spherical liposomes (Fig. 5A) were deformed into prolate ellipsoids (Fig. 5B) by increasing the temperature, stepwise, from −20 °C back to 25 °C, was submitted to a moderate spinning rate of 1.4 kHz (1400 rotor rotations per second), Fig. 5C at 25 °C. The pattern obtained was made of spinning side bands, spaced by 1.4 kHz, which map out the intensities of the initial powder pattern spectrum (Fig. 5A).

After stopping the sample rotation, a static ^31^P-NMR spectrum was acquired (Fig. 5D), showing the characteristics of a powder distribution, with *c/a ≈* 1.0 (simulation not shown). One has the clear evidence that magnetic field induced deformation could be destroyed by rapid rotation of the sample at the magic angle. Interestingly, the deformation phenomenon was reversible, *i*.*e*., the same field-induced prolate deformation could be obtained by submitting the sample to the same thermal variations in the magnetic field, without MAS. Cooling down to −20 °C and stepwise increase in temperature to 25 °C lead to the oriented like spectra of Fig. 5B (not shown). We may wonder why such a thermal “history” favors deformation. To explain this, we need to appreciate that the system goes through a kind of phase transition between low and high temperatures, which may aid to release membrane stress. At the phase transition there is a heterogeneity of phases that is certainly conducive to deformation, in a strong magnetic field.

## Discussion

The purification of lipids from mammalian nuclear envelopes and their reconstitution into liposomes has brought several new and interesting findings that can be summarized as follows. 1) The phospholipid composition is dominated by phosphatidylcholine, with remarkable amounts of negatively charged lipids and cholesterol. 2) Reconstituted nuclear membrane vesicles are fluid at ambient temperatures and enter into a gel-like solid ordered phase below 0 °C. Their membrane is more ordered (more rigid) than that of classical POPC membranes. 3) Reconstituted nuclear membrane vesicles are greatly deformed by magnetic field suggesting a highly deformable lipid membrane. These findings will be discussed sequentially and finally related to the propensity of large membrane invaginations that are observed for nuclear membranes.

The lipid composition that was analyzed by liquid state ^1^H, ^13^C and ^31^P-NMR shows that PC lipids represent more than 60 mol% of the phospholipid composition. Of interest, negatively charged lipids are present in large proportions, including phosphatidylinositol, PI, is ca. 15%. The presence of large amounts of PC lipids clearly accounts for the easy formation of micrometer-size liposomes when the lipid extract is rehydrated. It is known, after Isaelashvili^29^, that PC lipids have, in average, a cylindrical shape that favors the flat lamellar topology at the nanometer scale and hence the stabilization of bilayer membranes. ^1^H-NMR also indicates the presence of numerous unsaturation, which account for a low lamellar gel-to-fluid phase transition temperature (*vide infra*). It is interesting to compare our results to those of sea urchins nuclear membrane extracts^30,31^. In this model there are  three nuclear envelope membrane precursors, MV1, MV2 and NER. MV1 and NER are precursor membranes that are highly enriched rich in PI. The nuclear envelope remnants (NER) are rich in cholesterol. These nuclear envelope precursors are responsible for the initiation of nuclear envelope formation during the male pronucleus formation. Their lipid composition globally resembles the male pronucleus membrane precursors with a small variance in the quantity of PI lipids that appears in slightly lesser amount. It is however worth mentioning that non-silanized glassware was used in our study which may tend to minimise the detection of negatively charged species. Our results show that the hNM is enriched in PC, PE, PI and cholesterol and suggest that the NE lipid composition is globally similar to ER (endoplasmic reticulum) lipid membrane composition^32,33^. The presence of cholesterol is similar to the high levels of cholesterol in NER of the sea urchin^10^. The membrane domain is located at the acrosomal and centriolar fossae of the sperm and remains during the formation of the male pronucleus formation^34^. These are the membranous regions that have a role in chromosomal organization. We suggest the resemblance of the lipid composition, especially in the cholesterol levels, in sea urchin NERs and the NRs may lead to the similarity of their function in the chromosomal organization both in non-somatic and somatic cellular models. The outer nuclear membrane (ONM) is continuous with the ER^35^ therefore it is not too surprising finding similar lipid compositions. As Larijani and coworkers have shown that unsaturated fatty acid chained phosphoinositides are promoters of membrane disordering, their detection in mammalian nuclear membranes suggests favorably very dynamic membranes^10^.

Depending on their function, natural membranes may show very different thermotropic properties. It is recognized that the lipid membrane may be in a certain state of fluidity to perform its function^23,36^. For instance, the transition from an ordered state to a more disordered one in human erythrocytes occurs at *ca*. 20 °C^37^. On the other hand *Mycoplasma Laidlawii* membranes and lipid extracts show transition temperatures near 40 °C^38^ whereas *E*. *Coli* lipid extracts have been shown to transit from 20 to 40 °C depending on the fatty acid that was supplemented in the growing medium^39^. Extremophile *Archaea* grow at very high temperature, near very hot water sources, and show to be rather fluid above 80-90 °C^40^. Our finding concerning the reconstituted nuclear membrane vesicles indicate that the gel-to-fluid phase transition occurs near −10 °C and has a breadth of ca. 20 °C. Even at the lowest temperature investigated, −20 °C, the system is not completely in the solid-ordered state. This is remarkable and bound to the presence of many charged phospholipids and of unsaturation in the hydrocarbon chains. A more complete determination of chain length and unsaturation per lipid species is currently under way by mass spectrometry in our laboratories.

In addition to having a low transition temperature, reconstituted lipid nuclear membrane vesicles have been found to be slightly thicker than pure POPC membranes at ambient temperatures and up to 40 °C. We suggest that this is related to the presence of cholesterol in the lipid composition. Sterols are known to increase membrane ordering in the fluid phase^11,27^. The membrane ordering and thickness are however much less important than in membranes containing large amounts of sterols (30% and above), such as the plasma membrane. This suggests than the reconstituted nuclear membrane vesicles have increased stability but remain extremely fluid. We propose that the fluidity is bound to the presence of invaginations in the nuclear membranes, a more fluid membrane will help in stabilizing these large nuclear membrane deformations that can penetrate deeply in the nucleus (see below). To compare directly these results with our previous work^10^ on the formation of the male pronucleus in the sea urchin egg is not possible. The fluidity of the intact male pronucleus in the sea urchin egg is too complex to determine directly. Previously, we have used cell free system from the sea urchin model to measure the lipid composition and membrane dynamics of the nuclear envelope membrane precursors, such as NER and MV1. NERs have an elevated cholesterol and polyphosphoinositide composition and MV1 has an atypical polyphosphoinositide composition. Both these membranous subcompartments were very fluid as in the case of human nuclear membrane.

One of major findings in our work is that the reconstituted nuclear membrane vesicles of initial spherical shape can be remarkably deformed by a high magnetic field (18.8 T) into elongated ellipsoidal prolates. Magnetic alignment of proteins or lipids has already been reported^41–44^; it is in general very small as biological molecules have a very weak magnetic susceptibility, χ. However, molecules and cells may contain paramagnetic species, like iron ions, of high χ that will allow behaving as small orienting magnets. Lipid magnetic susceptibility anisotropy, Δχ, is in general very small (−10^−7^, dimensionless) and so lipids do not show orienting properties in solution. However, lipids under the form of liposomes may present cooperative orienting properties, the bulk magnetic susceptibility being the sum of the molecular susceptibilities. Seelig^44^ has first reported that liposomal lipid extracts could be oriented in magnetic fields, and Helfrich^28^ had long ago worked out the physics of magnetic-induced deformation of liposomes: under thermal equilibrium, liposomes are spherical in average and may be deformed when the magnetic orienting energy is greater than the membrane curvature elastic energy. Spherical liposomes are deformed into prolate or oblate ellipsoids, of semi axes *c* and *a*. The extend of deformation is expressed as *c-a* and energy minimization leads to, in the SI system (see supplementary information):

1 $$c-a\approx -f\frac{{r}_{0}^{3}\Delta \chi b{B}_{0}^{2}}{{\mu }_{0}{k}_{C}}$$

Where *r*_0_ (in m) is the initial radius of spherical liposomes, *b* (in m), the bilayer thickness, *B*_0_ (in Tesla, T = kg.s^−2^.A^−1^), the magnetic field intensity, *k*_*C*_ the apparent elastic energy modulus (in Joules, J = kg.m^2^.s^−2^) and $\Delta \chi ={\chi }_{\parallel }-{\chi }_{\perp }$, the anisotropy (dimensionless) of the magnetic susceptibility of molecules in the membrane. *f* and *μ*_0_ are constants of value 1/18 and 4π×10^−7^ (in kg.m.s^−2^.A^−2^), respectively. Δ*χ* is in general negative, e.g. −4.10^-7^ for dimyristoylphosphatidylcholine, (DMPC)^45^, for lipids having saturated or unsaturated chains. This will lead to *c-a* > 0 and to a prolate deformation as observed for nuclear lipid extracts. From the spectral simulation *c/a* was found close to 3.0 and calculating *c-a*, a value of 0.63 μm was obtained, which represents an substantial deformation of the initial spheres of radius *r*_0_ = 0.45 μm. In Eq. (1) all variables are known or measurable, except for Δ*χ* and *k*_*C*_. In other words, the deformation scales as $\,\Delta \chi /{k}_{C}$ and may increase because the membrane elastic modulus (of the order of 10^-19^ J) decreases or the anisotropy of the membrane magnetic susceptibility, Δ*χ*, increases. Δ*χ* values are very difficult to determine and have been scarcely measured up to now; there are debates about the accuracy in their determination. In case of lipid mixtures, it will be the weighed sum of the molecular magnetic susceptibilities of each of the lipid species. Borowske & Helfrich^45^ nonetheless reported a value for egg lecithin membranes: −0.28.10^-8^ cgs units, which translates in the SI system as $\Delta {\chi }_{SI}=4\pi \Delta {\chi }_{CGS}$ = −3.5.10^−7^. By making the rough hypothesis that the magnetic susceptibility anisotropy in reconstituted human nuclear membrane vesicles and in egg lecithin are similar one may calculate the corresponding elastic modulus: *k*_*C*_ ≈ 0.03.10^−19^ J. This value may be compared to some reliable values for DMPC in the presence of various amounts of cholesterol^46^, *k*_*C*_ (DMPC) = 1.27.10^−19^ J and *k*_*C*_ (DMPC + 30 mol% CHOL) = 3.07.10^−19^ J. It therefore appears that the membrane elasticity modulus of nuclear membranes is very small meaning that membrane undulations are very much favored. Figures seem to indicate about 2 orders of magnitude more elastic membranes compared to plasma-like membranes. Of course, this conclusion must be taken with care as we hypothesized a similar magnetic susceptibility for nuclear lipid membranes and egg lecithin membranes. However, the difference in elasticity is so elevated that it cannot be compensated by large variations in Δ*χ*. It is worth mentioning here that there are many other techniques to measure elastic modulii: Evans’ micropipette technique^47^ or Faucon’s video microscopy^48^ on giant vesicles or Epand’s by X-Rays^49^, or ourselves using NMR and X-rays on water-swelling systems^50^. All these methodologies require the use of Giant vesicles of at least 10 mm diameter that are very hard to form especially with natural lipids or use indirect measurements in hexagonal phases swollen with polymers. Our method is non-invasive and by far simpler: liposomes at rest placed in high magnetic fields.

The most striking result of our study is the finding of very fluid and highly deformable nuclear lipid membrane. To our knowledge this is the first time that such measurements of physical parameters have been made. We believe that their special lipid composition, PC lipids, charged lipids, including PI lipids, cholesterol and a high concentration of chain unsaturation is at the origin of such phenomena. The elasticity of lipid nuclear membranes is much more important than that usually reported for plasma lipid membranes. This indicates that the nuclear lipid membrane bears this intrinsic property that would lead to large membrane fluctuations (undulations, hydrodynamic deformations, etc.), in the absence of membrane proteins and lamina proteins present in the nucleus. The time scale that is usually reported for large scale membrane undulations ranges from milliseconds to several seconds. Our results can be linked with the fact that NE has to promote fusion events and/or nuclear invaginations. Even if these results are based on natural membrane lipids without proteins we may suggest that a such lipid composition is one of the requirements to promote the formation of nuclear invaginations, that is to say, the lipid matrix has the intrinsic property to deform almost at will. Of course, such a dynamic membrane has to be bound to other proteins to prevent unwanted undulations; invaginations would be the result of lipid membrane plasticity and special protein anchoring. Although, nuclear invagination functions are still under investigation, there is primarily evidence of their involvement in calcium signaling, gene expression and transport^51–54^. Furthermore, it has been shown that lipid synthesis and modification enzymes occurs within nuclei, together with independent nuclear phosphoinositide and diacylglycerol pools^51–54^. In addition, it has been reported that the morphology of nuclear invaginations can change on a timescale of 5 minutes^55^, a time scale that is close to that observed for hydrodynamics modes for membrane undulations reported herein. Such a dynamic structure suggests that its formation is regulated and that the mechanisms of regulation could be linked to nuclear invaginations function and composition. From our results, we propose that the lipid composition of the nuclear envelope is involved in the formation of such substructures. It has also been reported that in some pathologies the morphology of the nuclear invaginations is altered suggesting the importance of their lipid composition. We propose that the dysregulation of the lipid pathways, maintaining the correct lipid compositions, may lead to various pathologies.

## Materials and Methods

### Chemicals

Cell culture reagent, Dulbecco’s Modified Eagle’s Medium (DMEM), Foetal Bovine Serum (FBS) and Streptomycin/Penicillin were purchased from Invitrogen, (Carlsbad, CA. USA). ^2^H_31_ palmitoyl, 2-oleoyl-*sn*-glycero-3-phosphocholine (POPC-^2^H_31_) was purchased from Avanti Polar Lipids (Birmingham, AL. USA). These starting materials were used without further purification. Deuterium-depleted water was obtained from Eurisotop (Saint Aubin, France) and solvents for liposome preparation (chloroform, methanol and ethanol) were obtained from Sigma Aldrich Chemicals (Saint Quentin Fallavier, France).

### Human nuclear membranes purification

Nuclei purification without use of detergent was performed following a protocol that we optimized from Blobel & Potter^16^. Details will be published elsewhere. Typically, human Embryonic Kidney (HEK) 293 T cells were grown on tissue culture dishes in DMEM supplemented with FBS and Streptomycin/Penicillin). After harvesting by trypsinization, cells were incubated in a hypotonic buffer and then lysed by nitrogen cavitation in a cell disruption bomb (Parr Instrument Company, Il. USA). This method prevents alteration of membrane lipid composition as could happen using a classical detergent protocol. Nuclei were purified with a sucrose gradient. Nuclei were further labelled with DiOC_6_ (1 µM) and Hoechst 33343 (1.6 µM) and observed by confocal fluorescence microscopy to assess the efficiency of nuclei isolation.

### Fluorescence imaging microscopy

Cells and nuclei were placed on polylysine-coated MatTek dishes. (Cultureware, Ashland, MA. USA) and visualized under an inverted confocal microscope with a high-efficiency spectral detector (Leica TCS SP5; Leica Microsystems, Manheim, Germany). A 63-fold glycerol immersion, N.A. 1.3 objective was used, and the images were collected and analyzed with the LAS AF software (Leica Microsystems, Manheim, Germany). DiOC_6_ was excited at 488 nm using an argon laser and its emission was collected in the 504-661 nm range. Hoechst was excited at 405 nm using a pulsed diode laser and its emission was collected in the 283-436 nm range. All experiments were performed at room temperature (*ca*. 20 °C).

### Lipid extraction

Lipids were extracted from nuclei samples using a modified Folch extraction^31^. Nuclei were added to 4 mL of acidified chloroform:methanol (2.5:1), sonicated and filtered. After addition of 0.2 volumes of K_4_EDTA (0.2 M, pH 6), samples were centrifuged at 800 g. The lower phase was retained and dried down completely at 55 °C, under nitrogen gaz. Phospholipid concentration was determined by a Fiske assay resulting in an indirect measurement of inorganic phosphates released from extracted lipids^55^. Phospholipid characterization and quantification were provided by liquid-state ^31^P NMR as described below.

### Sample preparation for liquid-state NMR

4 mg of lipid extract dried under N_2_ were resuspended in milliQ (Millipore, Billerica, MA) filtered water, flash-frozen with liquid N_2_ and lyophilized overnight. The fluffy powder was dissolved into 450 µL of MeOD/CDCl_3_ (1:2). 30 µL of EDTA 0.2 M/D_2_O pH 6 was added to chelate residual paramagnetic ions^56^. The lipid extract solution (450 µL) was poured into a 5-mm diameter glass tube (Cortecnet, Voisins Le Bretonneux, France) together with a sealed 1 mm reference tube containing 50 µL of MeOD/CDCl_3_ (1:2) with 2 µmol of the internal standard TPP (Triphenylphosphate) for proton (^1^H) and Phosphorus-31(^31^P) NMR quantification.

### Sample preparation for solid-state NMR

0.8 mg of POPC-^2^H_31_ were dissolved into 80 µL of MeOH/CHCl_3_ (1:2) and added to 8 mg of nuclear lipid extract (molar ratio of ca. 1:10). The solvent was evaporated under a nitrogen gas flux. The residual lipid film was dispersed in 1 ml milliQ filtered water and lyophilized overnight. The resulting fluffy powder was suspended into 80 µL of a 10 mM HEPES buffer (5 mM MgCl_2_, pH 7.2, made with deuterium-depleted water) to obtain a hydration, h, of 90% (h = mass of water over the total mass of the system (phospholipids plus water)). After shaking into a vortex mixer samples were frozen in liquid nitrogen for 30 s, heated at 40 °C for 10 min in a water bath and shaken again for better sample homogeneity; this freeze-thaw-shaking cycle was repeated 3 times and the resulting milky dispersion transferred into a 4 mm diameter Zirconium rotor (80 μL, (Cortecnet, Voisins Le Bretonneux, France).

#### NMR spectroscopy

Liquid-state ^1^H- ^13^C- and ^31^P-NMR experiments were carried out on a Bruker Avance III-HD 400 MHz SB spectrometer (Wissembourg, France) equipped with a 5 mm broadband SmartProbe at 25 °C. ^31^P-NMR spectra were acquired at 161.98 MHz by using a one-pulse sequence with proton decoupling (π/2 pulse width of 8 μs, recycling delay of 10 s, acquisition time of 4 s, spectral window of 54 ppm and between 80 and 1024 summed aquisitions). ^1^H-NMR spectra were acquired at 400.13 MHz using a single pulse sequence (π/2 pulse width of 10 µs, recycling delay of 2 s, acquisition time of 2 s, spectral window of 20 ppm and 48 scans). Two-dimensional experiments were performed at 400.13 MHz using a HSQC-TOCSY 2D-sequence^57^ used to identify/assign chemical shifts for various phospholipids. The ^1^H-^31^P HSQC-TOCSY 2D-map was obtained with transfer delays corresponding to a 7 Hz proton-phosphorus coupling constant and an additional 50 ms DIPSI mixing period for protons after the HSQC step. The other parameters were a recycle delay of 2 s, ^1^H and ^31^P 90 π/2 pulse widths of respectively 10 and 8µs, acquisition time of 0.3 s, 48 scans, 9 and 10 ppm spectral widths in proton and phosphorus dimensions, respectively, 2 K data points for the F2 dimension and 176 data points for the F1 dimension. The ^1^H-^13^C HSQC (Heteronuclear Single-Quantum Correlation) experiment allows to obtain a 2D heteronuclear chemical shift correlation map between directly-bonded ^1^H and ^13^C. The polarization transfers are obtained via INEPT blocks and proton-carbon coupling constant of 145 Hz was used. The other parameters were a recycle delay of 1.5 s, ^1^H and ^13^C 90 π/2 pulse widths of respectively 10 µs for both, acquisition time of 0.14 s, 24 scans, 18 and 165 ppm spectral widths in proton and carbon dimensions, respectively, 2 K data points for the F2 dimension and 256 data points for the F1 dimension. Solid-state ^31^P and ^2^H NMR were performed on a Bruker Avance III 800 MHz SB spectrometer (Wissembourg, France) equipped with a dual H/X 4-mm MAS probe. ^31^P-NMR spectra were acquired at 323.96 MHz by using a proton decoupled Hahn-echo pulse sequence^58^. Typical acquisition parameters were as follows: spectral window of 200 kHz, π/2 pulse width of 5.4 µs, interpulse delay of 40 µs and recycle delay of 5 s. Typically 5 k scans were accumulated. Phosphorus chemicals shifts were calibrated relative to H_3_PO_4_ (85% in H_2_O, 0 ppm). Magic angle sample spinning (MAS) was accomplished on some samples, a spinning rate of 1.4 kHz was applied and the ^1^H-decoupling-one-pulse sequence used with the following parameters: ^31^P π/2 pulse width of 5.4 µs, recycling delay of 5 s, acquisition time of 40.9 ms, spectral window of 100 kHz and 100 scans, proton decoupling at 10 W power. ^2^H-NMR spectra were acquired at 122.82 MHz by means of a quadrupolar echo pulse sequence^59^, with a spectral width of 500 kHz, a π/2 pulse width of 4.5 µs, a 40 µs interpulse delay and a recycle delay of 2 s. Typically, 10-50 k scans were recorded depending on temperature. The reference for solid-state deuterium powder patterns was set to zero and the position of the carrier arbitrarily placed in the middle of the symmetric pattern. A Lorentzian noise filtering of 100–500 Hz was applied prior Fourier transformation from the top of the echo signal. Quadrature detection was used in all cases. Samples were allowed to equilibrate at least 30 min at a given temperature before the NMR signal was acquired.

### Optical microscopy and size of liposomes

POPC and reconstituted nuclear membrane vesicles were added in an 8-wells plate (Lab-Tek, Nalc Nunc International) diluted 3-fold in order to be observed by microscopy. Liposomes were let settle down for 24 h ensuring that all liposomes were in the same focal plane. DIC (Differential Interference Contrast) and Epifluoresence imaging were processed using an IX81 (Olympus) microscope. For Epifluorescence imaging, liposomes were stained by the dye FM 1-43 (N-(3-Triethylammoniumpropyl)-4-(4-(Dibutylamino) Styryl) Pyridinium Dibromide) diluted one hundred times. Particle size analysis was performed with the Image J software^60^ from DIC images obtained with a 60 fold oil objective. A size histogram was constructed based on 4 DIC images containing a total of 3000–3600 objects. A gauss function was used within the Origin Pro 9 Software (OriginLab, Northampton, MA) in order to obtain the mean diameter of vesicles. Mean diameters are 0.9 ± 0.4 µm for reconstituted nuclear membrane vesicles and of 1.0 ± 0.4 µm POPC vesicles (Supplementary Fig. 4).

### NMR data treatment and spectral simulations

Liquid state ^1^H-, ^13^C- and ^31^P-NMR spectra were processed using the Bruker TopSpin software. All peaks in 1D-^31^P NMR spectra were integrated and converted into molar quantities by comparison with the internal standard triphenylphosphate (TPP), and the whole spectrum was integrated to obtain the total quantity of Phospholipids (see Supplementary Fig. 1). The quantity of each of the phosphorus-containing lipid species was determined by simulating ^31^P liquid-state NMR spectra with the DMFIT software^61^. 2D-NMR maps were used as phospholipid finger prints to confirm their chemical shift assignment. Identification was based on head group ^1^H-^31^P cross peaks according to literature^61^. For wide-line solid-state NMR, spectral moments allow to quantitate spectral changes as function of temperature and were calculated according to Dufourc^23^ and Davis^20^ using a FORTRAN code developed by Erick Dufourc and implemented in a user-friendly routine, NMRFriend, by Sébastien Buchoux^62^. Wide-line solid-state NMR spectra were simulated by calculation in the time domain (as free induction decays) and then Fourier transformed. Individual components are built from experimental estimates of chemical shielding anisotropies (Δσ) or quadrupolar splittings (Δν_Q_), isotropic chemical shifts and individual line-widths (line-width is considered constant throughout the pattern). Small variations are allowed to match with sharp experimental features on spectra. For lipids containing perdeuterated chains, weights for individual C^2^H_2_ or C^2^H_3_ depend on the number of deuterons per labelled carbon position; the individual time dependent signals are then added accordingly leading after Fourier transformation to the multicomponent spectrum. Such a simulation leads to individual quadrupolar splittings, $\Delta {\nu }_{Q}^{k}$, for labelled carbon positions, k, and subsequently to *S*^*CD*^ order parameters in bilayer membranes^20^: ${S}_{k}^{CD}=4\Delta {\nu }_{Q}^{k}/3{A}_{Q}$. Order Parameters can be used to calculate the average length of a lipid molecule in terms of a sum of chain, glycerol backbone and head-group average lengths^26^: ${L}_{lipid}={L}_{chain}+{L}_{gly}+{L}_{head}$ (See supplementary information, Table 1). Liposome deformation leading to non-spherical distributions of bilayer normals with respect to the magnetic field was taken into account by introducing in the simulation the ellipsoidal orientation dependence, $p(\theta ) \sim \frac{si{n}^{2}\theta }{si{n}^{2}\theta +\frac{c}{a}co{s}^{2}\theta }$, where *θ* is the orientation of bilayer normal with respect to the magnetic field direction and *c* and *a* the ellipsoid axes^27,28^. The simulation program for wide line spectra has been developed in FORTRAN code by Erick Dufourc and implemented in a user-friendly graphical interface (Microsoft.NET) for Windows platforms by Arnaud Grélard. The program is available on demand.

## Supplementary information

Supplementary information.
