# Supplementary material for: The unprecedented membrane deformation of the human nuclear envelope, in a magnetic field, indicates formation of nuclear membrane invaginations
Source: Sci Rep. 2020 Mar 20;10:5147. doi: 10.1038/s41598-020-61746-0 (PMC7083927; doi:10.1038/s41598-020-61746-0)
Supplement: Supplementary file 1 — Supplementary information. [file 41598_2020_61746_MOESM1_ESM.docx]

***Supplementary Information***

The unprecedented membrane deformation of the human nuclear envelope, in a magnetic field, indicates formation of nuclear membrane invaginations

Régine Dazzoni^1,2^, Axelle Grelard^1^, Estelle Morvan^3^, Anthony Bouter^1^, Christopher J. Applebee^2^, Antoine Loquet^1^, Banafshé Larijani^2*^, Erick J. Dufourc^1*^

^1^Institute of Chemistry & Biology of Membranes & Nanoobjects, UMR5248, CNRS, Université Bordeaux, Institut National Polytechnique Bordeaux, F-33600 Pessac, France.

^2^Cell Biophysics Laboratory, Ikerbasque Basque Foundation for Science, Instituto Biofísika (CSIC, UPV/EHU) and Research Centre for Experimental Marine Biology and Biotechnology (PiE), University of the Basque Country (UPV/EHU), Spain

^3^Institut Européen de Chimie et Biologie , University of Bordeaux, INSERM, CNRS (UMS3033- US001), 2 rue Escarpit, Pessac 33600, France

* Correspondence should be addressed to Erick J. Dufourc ([e.dufourc@cbmn.u-bordeaux.fr](mailto:e.dufourc@cbmn.u-bordeaux.fr) or erick.dufourc@cnrs-dir.fr), to Antoine Loquet (a.loquet@iecb.u-bordeaux.fr) or to Banafshé Larijani (banafshe.larijani@ikerbasque.org)

Contents

Supplementary Figures 1 to 6

Supplementary Tables 1 to 2

Theory of magnetically induced liposome deformation

Considerations on magnetic, curvature elastic and mechanic energies


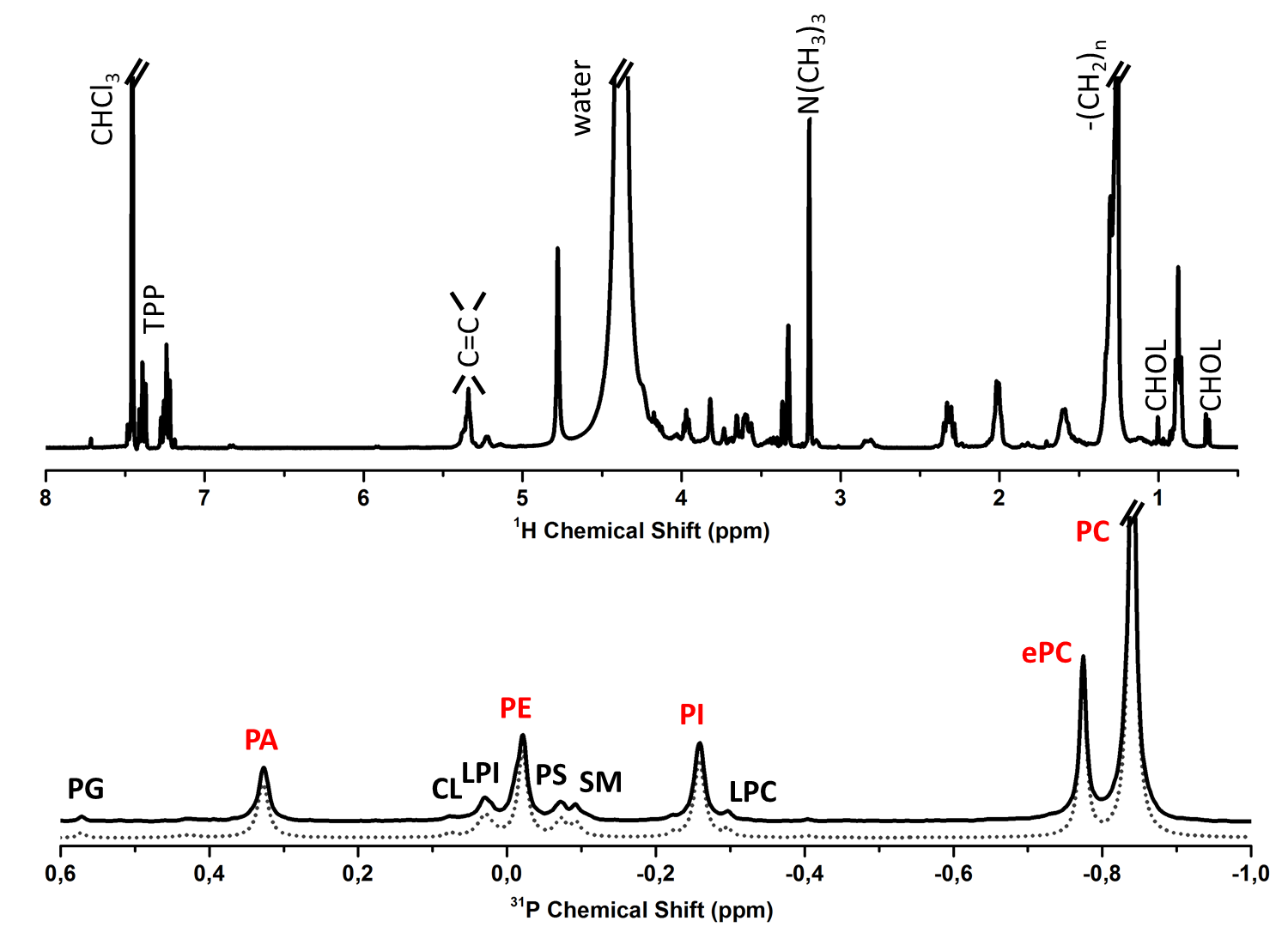


Supplementary Fig. 1. **NMR quantification of phospholipids in human nuclear membranes.** Liquid-state ^31^P-NMR (bottom) & ^1^H-NMR (top) spectra of human Nuclear Membrane Lipids corresponding to 4 mg of phospholipids dissolved in chloroform-methanol 2:1, 0.2M EDTA-D_2_O, pH 6. Phospholipids assignments were based on ^31^P chemical shift from Meneses & Glonek and Kaffarnic *et al.*^1,2^, and also from ^1^H-^31^P 2D experiments (Fig. 2 below). TPP: triphenylphosphate (external reference) was used as a standard for chemical shift and concentration (see main text), PC: phosphatidylcholine, EPC: ether phosphatidylcholine, LPC: lysophosphatidylcholine, PI: phosphatidylinositol, LPI: lysophosphatidylinositol SM: sphingomyelin, PS: phosphatidylserine, PE: phosphatidylethanolamine, CL: cardiolipin, PA: phosphatitic acid, PG: phosphatidylglycerol. Red labels stand for secured assignment using 2D NMR and black labels represent assignments by comparison with literature. ^31^P acquisition conditions: number of scans = 1024, recycling delay = 10s, Lorentzian filtering = 1 Hz. The dotted line below the experimental phosphorus spectrum represents the simulated spectrum using the DMFIT software ^3^. Peak lineshape was approximated by a Gaussian line to account for experimental inhomogeneity (water dispersions). The area below each peak is reported in Table 1 of main text as a percentage of the total area. On the proton spectrum (top), labels stand for straightforward chain, chain unsaturation (5.3 and 5.4 ppm) and head group assignment using literature, CHOL labels highlights characteristic CH_3_ resonances of cholesterol (further evidences for cholesterol resonances are see in the 2D spectrum of Fig. 3). Number of scans: 48 with a recycling delay of 2 s and a Lorentzian filtering of 0.3 Hz. Very intense peaks were cut in the vertical expansion to show details on lower intensity peaks.


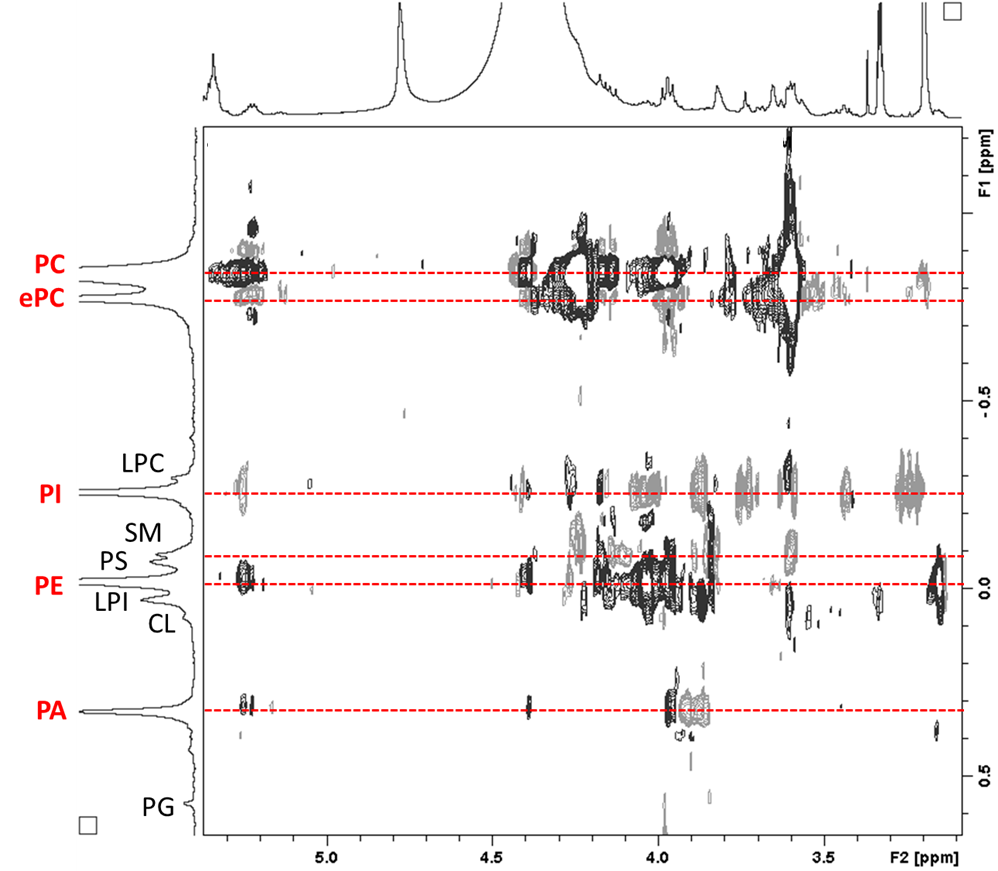


Supplementary Fig. 2. **Assignment of lipid species.** Liquid-state ^1^H-^31^P HSQC-TOCSY 2D map of Nuclear Membrane Lipids corresponding to 4 mg of phospholipids dissolved in chloroform-methanol 2:1, 0.2M EDTA-D_2_O, pH 6. The transfer delay was adjusted to correspond to a 7 Hz average proton-phosphorus coupling constant. The other parameters were a recycle delay of 2s, ^1^H and ^31^P 90 π/2 pulse widths of respectively 10 and 8µs, acquisition time of 0.3s, 48 scans, 9 and 10 ppm spectral widths in proton and phosphorus dimensions, respectively, 2K data points for the F2 dimension and 176 data points for the F1 dimension.

PC: phosphatidylcholine, EPC: ether phosphatidylcholine, LPC: lysophosphatidylcholine, PI: phosphatidylinositol, LPI: lysophosphatidylinositol SM: sphingomyelin, PS: phosphatidylserine, PE: phosphatidylethanolamine, CL: cardiolipin, PA: phosphatitic acid, PG: phosphatidylglycerol. Red labels stand for secured assignment using this map and black labels represent assignments by comparison with literature ^1,2,4,5^.


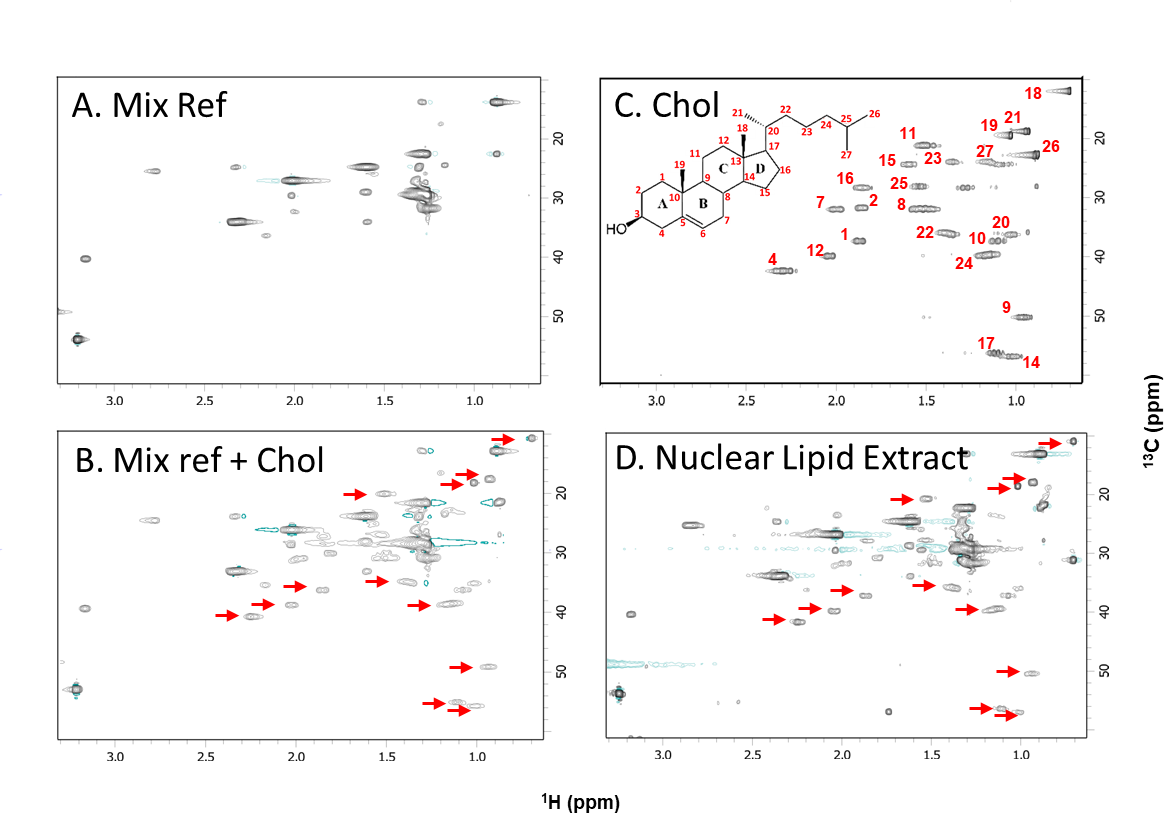


Supplementary Fig. 3: **Identification of cholesterol in the Nuclear Lipid Extract.**

A) ^1^H-^13^C HSQC 2D experiment of a mixture of phospholipid references (PG: 8 % mole, PA: 8 %, CL: 6 %, PE: 18 %, SM: 4 %, PS: 5 %, PI: 6 %, LPC: 4 %, PC: 41 %) dissolved in CDCl_3_:CD_3_OD. B) ^1^H-^13^C HSQC 2D experiment of the same mixture of phospholipid references with cholesterol (ca. 20% with respect to the total phospholipid amount). Red arrows highlight cholesterol resonances on comparing with Fig. 3C. C) ^1^H-^13^C HSQC 2D experiment on cholesterol dissolved in CDCl_3_:CD_3_OD; insert shows its chemical structure with carbon numbering; assignment of cholesterol ^1^H-^13^C correlations (numbers corresponding to carbons) was based on the SDBSweb^6^ D) ^1^H-^13^C HSQC 2D experiment of 4 mg of the nuclear lipid extract. Red arrows highlight cholesterol resonances on comparing with Fig. 3C and 3B.

Acquisition parameters: The transfer delays were set to correspond to 145 Hz as an average for the proton-carbon coupling constant. The other parameters were a recycle delay of 1.5s, ^1^H and ^13^C 90 π/2 pulse widths of respectively 10µs for both, acquisition time of 0.14s, 16 to 96 scans depending on the concentration of each sample, 18 and 165 ppm spectral widths in proton and carbon dimensions, respectively, 2K data points for the F2 dimension and 256 data points for the F1 dimension.


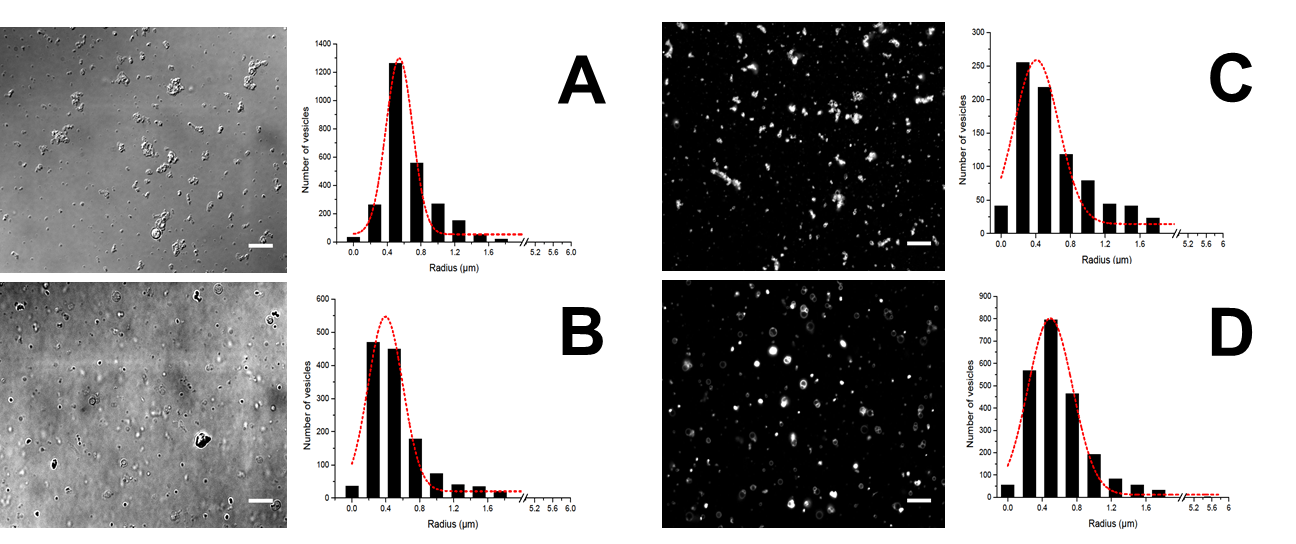


Supplementary Fig. 4. **Size determination for Nuclear Lipid Extract (NLE) Vesicles and POPC MLV**. POPC vesicles (A/C) and NLE vesicles (B/D) as used for solid state NMR experiments were diluted 1000 times in a HEPES buffer (10 mM, 5mM MgCl_2_, pH7.2). Images were obtained by Differential Interference Contrast (DIC), (A/B), and epifluorescence, (C/D), microscopy with a magnification x 60 after staining vesicles with the FM1-43 probe (5ng/µL). Vesicles radii were obtained using the Image J software and size distributions are reported on the right-hand side of the corresponding image. A Gaussian fitting (dotted red line) was performed using OriginPro Software. 3000 and 3600 vesicles were respectively analysed for POPC and NLE respectively; mean diameters are 0.9±0.4 µm for NLE vesicles and of 1.0 ±0.4 µm for POPC vesicles.

Supplementary Fig. 5. **Thermal variation of deuterium solid-state NMR spectra.** ^2^H-NMR spectra of reconstituted nuclear lipid membrane extracts (NLE) vesicles doped with 10% deuterated POPC during a thermal variation (25°C down to -20°C and back, from left top to right bottom) and recorded after temperature stabilization. Spectra were obtained after Fourier transformation of solid-echo type experiments accumulated for 10 to 50 k transients, Lorentzian line filtering of 100 to 500 Hz. Details for experimental parameters and data treatment are found in the methods section. The lower right spectrum (asterisk) was obtained after spinning the sample at the magic angle (1.4 kHz) and let the system stabilize at 25°C after stopping MAS.


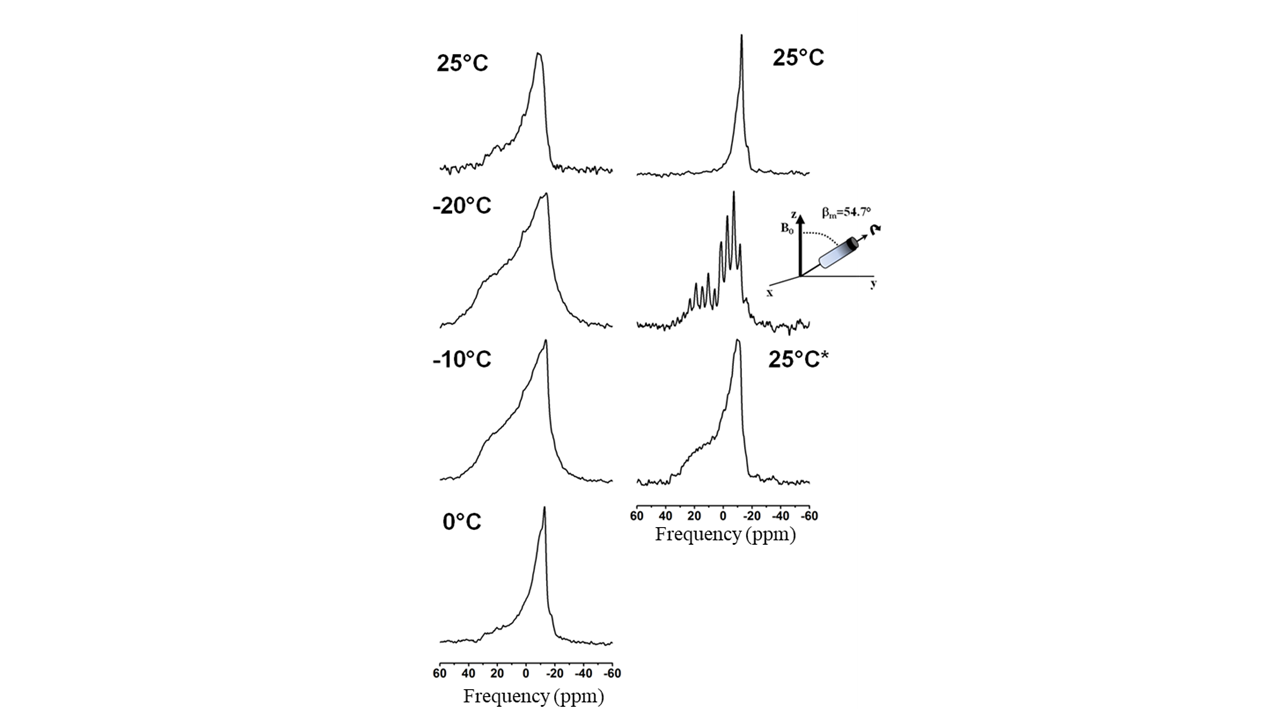


Supplementary Fig. 6. **Thermal variation of ^31^P-NMR spectra.** ^31^P-NMR spectra of reconstituted nuclear lipid membrane extracts (NLE) during a thermal variation (25°C down to -20°C and back, from left top to right top) and recorded after temperature stabilization. Spectra were obtained after Fourier transformation of Hahn-echo type experiments accumulated for 0.3 to 7k transients, Lorentzian line filtering of 300 Hz. Details for experimental parameters and data treatment are found in the methods section. Middle spectrum on right column was acquired at 25°C under magic angle sample spinning at 1.4 kHz and shows a spinning side band pattern picturing the “powder-like” pattern (*i.e.*, destruction of magnetic alignment as seen on the above spectrum). The insert aside the spectrum depicts the MAS set up, the rotor spinning at an angle of 54.7° with respect to the magnetic field. The lower right spectrum (asterisk) was obtained after spinning the sample at the magic angle (1.4 kHz) and let the system stabilize at 25°C after stopping MAS.

Supplementary Table 1. **Chain order parameters from ^2^H-NMR spectra**. Experimental quadrupolar splittings, order parameters and bilayer thickness calculation for NLE containing ^2^H_31_-POPC and for pure ^2^H_31_-POPC liposomes, at 25°C.

|  | NLE | | | POPC | | |
| --- | --- | --- | --- | --- | --- | --- |
| #C | *Δν_Q_^a^* | *S_CD_^b^* | *S_CC_^c^* | *Δν_Q_^a^* | *S_CD_^b^* | *S_CC_^c^* |
| 2 | 29.60 | -0.236 | 0.230 | 25.60 | -0.204 | 0.195 |
| 3 | 29.60 | -0.236 | 0.242 | 25.60 | -0.204 | 0.213 |
| 4 | 29.60 | -0.236 | 0.230 | 25.60 | -0.204 | 0.195 |
| 5 | 29.60 | -0.236 | 0.242 | 25.60 | -0.204 | 0.213 |
| 6 | 29.60 | -0.236 | 0.230 | 25.60 | -0.204 | 0.195 |
| 7 | 29.60 | -0.236 | 0.242 | 25.60 | -0.204 | 0.213 |
| 8 | 27.10 | -0.216 | 0.230 | 22.65 | -0.180 | 0.195 |
| 9 | 27.10 | -0.216 | 0.201 | 22.65 | -0.180 | 0.166 |
| 10 | 25.45 | -0.203 | 0.230 | 21.30 | -0.170 | 0.195 |
| 11 | 22.75 | -0.181 | 0.175 | 18.90 | -0.151 | 0.145 |
| 12 | 20.50 | -0.163 | 0.187 | 16.95 | -0.135 | 0.156 |
| 13 | 17.50 | -0.139 | 0.139 | 14.55 | -0.116 | 0.114 |
| 14 | 14.50 | -0.116 | 0.140 | 12.25 | -0.098 | 0.118 |
| 15 | 10.70 | -0.085 | 0.091 | 9.15 | -0.073 | 0.077 |
| 16 | 3.05 | -0.024 | 0.079 | 2.65 | -0.021 | 0.069 |
| S_mol_^d^ | 0.75 | | |  | 0.7 | |
| L_chain_^e^ | 13.7 | | |  | 13.0 | |
| b^f^ | 44.2 | | |  | 42.8 | |

^a^Obtained from spectral simulations of figure 2A of main text and represents the splitting for orientations of bilayer normals at 90° to the magnetic field, accuracy is better than 0.5 %. Assignment was based on literature ^7,8^. Positions 2-9 could not be separated.

^b^Calculated from *Δν_Q_* using the equation ${\Delta\nu}_{Q}^{k}=\frac{3}{4}A_{Q}S_{CD}^{k}$ from ^9^ with *A_Q_* = 167 kHz ^10^. Accuracy is 0.5 %.

^c^Calculated from $S_{k}^{CD}$ using the recurrent equation^11^: ${2S}_{k}^{CD}=-(S_{k}^{CC}+S_{k+1}^{CC})$, accuracy is 0.5%. $S_{16}^{CC}$is obtained using the C3 symmetry around the C_16_-C_15_ bond: $S_{16}^{CC}=S_{16}^{CD}/(\frac{{3cos}^{2}111^{\circ}-1}{2}))$

^d^*S_mol_*, the molecular order parameter was obtained from ^7,8^ for DMPC and DMPC + 30% cholesterol. Value for NLE were extrapolated by considering a maximum of 20% cholesterol in the bilayer.

^c^*L_chain_* was calculated from ^7,8^. $\left\langle L_{chain} \right\rangle=\frac{1+\sqrt{1+8S_{mol}}}{4}\left[ \left\langle l_{C_{n}-H} \right\rangle+1.25\sum_{k=2}^{n} \left( \frac{1}{2}+\frac{S_{k}^{CC}}{S_{mol}} \right) \right]$, where $\left\langle l_{C_{n}-H} \right\rangle$ was the contribution of the methyl terminus (0.81 Å).

^f^Under the assumption ^7,8^ that there were no lipid interdigitation, the bilayer thickness was obtained by summing two tail-to-tail molecular lengths: b = 2$\left\langle L_{lipid} \right\rangle$, where $\left\langle L_{lipid} \right\rangle=\left\langle L_{chain} \right\rangle+\left\langle L_{gly} \right\rangle+\left\langle L_{head} \right\rangle$. $\left\langle L_{lipid} \right\rangle=\left\langle L_{chain} \right\rangle+\left\langle L_{gly} \right\rangle+\left\langle L_{head} \right\rangle$. $\left\langle L_{head} \right\rangle$ + $\left\langle L_{gly} \right\rangle$ = 8.4 Å was obtained by combining neutron diffraction data for the lipid length, 21.8Å, and NMR data for the chain length, 13.4 Å, calculated for DPPC in the fluid phase. Accuracy for *L_chain_* is ± 0.4Å. Accuracy for *b* is estimated to be ±1Å.

Supplementary Table 2. **Simulations of ^31^P-NMR spectra.** Experimental chemical shielding anisotropies and deformation parameters were obtained from simulation of ^31^P-NMR spectra, at 25°C.

|  | NLE | | | POPC | | |  |
| --- | --- | --- | --- | --- | --- | --- | --- |
|  | $\Delta\sigma$ *^a^* | | weight^b^ | $\Delta\sigma$ *^a^* | | weight^b^ | $\Delta\sigma$*^c^* |
| Assignment^d^ | kHz | ppm | % | kHz | ppm |  | ppm |
| PI | 15.6 | -48.3 | 11±3 |  |  |  | -61 |
| PS | 14.6 | -45.2 | 1±3 |  |  |  | -50 |
| PC | 11.5 | -35.7 | 66±3 | 15.00 | -46.4 | 100 | -47 |
| PE | 8.8 | -27.3 | 12±3 |  |  |  | -43 |
| PA | 7.2 | -22.3 | 9±3 |  |  |  | -50 |
| CL | 5.4 | -16.7 | 1±3 |  |  |  | -33 |
| *c/a*^e^ | 3.0 | |  | 1.0 | | |  |

^a^Chemical Shielding anisotropy, ${\Delta\sigma=\sigma}_{//}-\sigma_{\perp}$, acquired at a Larmor frequency of 323 MHz, accuracy ±1%.

^b^Weigth of each pattern, accuracy ± 3%

^c^Literature values from isolated species: PC, PE, CL values at 50°C from Shin ^12^, PS value at 30°C from Tilcock & Cullis ^13^, PA value at 5°C, pH 7 from Cullis ^14^, PI value at 10°C from Zhendre ^15^. It has been shown that the presence of PI and cholesterol promotes a decrease in the chemical shielding anisotropies of phospholipids ^16,17^

^d^Tentative assignment using literature values and Table 1 of main text.

^e^Anisotropy ratio (*c* = long axis, *a* = short axis) defining the prolate ellipsoid, accuracy ±0.2

**Theory of magnetically induced liposome deformation**

Following the Helfrich theory ^18,19^ one may describe the magnetically induced deformation of membrane bilayer spheres of initial radius *r_0_* (at equilibrium, outside magnetic field) to ellipsoids of revolution of semi-long and semi-short axes *c* and *a,* respectively, as the combined effects of both the magnetic energy and the membrane elastic energy.

Scheme S1. **Bilayer spheres of radius *r_0_* (μm) deform into ellipsoids of revolution upon magnetic field action**. *c* and *a* are the semi-long and semi-short axes*,* respectively (expressed in μm). The bilayer thickness is denoted *b* (nm) and the magnetic field *B_0_* (T). $\chi_{\parallel}$ and $\chi_{\perp}$ are the magnetic susceptibilities for magnetic field normal (parallel) and tangential (perpendicular) to the bilayer plane.

The deformation happens when the membrane curvature-elastic energy, *E_C_*, is smaller than the orientation energy in the magnetic field, *E_H_* ^18,19^:

$$E_{C}= \pi k_{C}\left[ 2\left( 2-r_{0}c_{0} \right)^{2})+\frac{\frac{9}{4}\left( c-a \right)^{2}}{r_{0}^{2}}\left( \frac{48}{5}-\frac{8\left( r_{0}c_{0} \right)}{5} \right) \right]$$

$$E_{H}=- \pi\Delta\chi b\frac{B_{0}^{2}}{\mu_{0}}\left[ \left( \frac{2}{3}r_{0}^{2}-\frac{8r_{0}\left( c-a \right)}{5} \right) \right]$$

Where $k_{C}$ is the membrane curvature-elastic modulus (in J), *B_0_* the magnetic field induction (in T) and $\Delta\chi=\chi_{\parallel}-\chi_{\perp}$, the anisotropy of the magnetic susceptibility of molecules in the membrane. Minimization in energy leads to deformation into an ellipsoid expressed as (SI System):

$$c-a\approx- f\frac{r_{0}^{3}\Delta\chi bB_{0}^{2}}{\mu_{0}k_{C}}$$

Where *f* = 1/18 for symmetric bilayers (*c_0_* = 0) and *μ_0_* is the vacuum permeability = 4π×10^−7^ (in N/A^2^ or kg.m.s^−2^.A^-2^).

As the anisotropy ratio *c/a* can easily be obtained from NMR simulations (*vide supra*) and by considering that there is no significant variation of the liposome volume under deformation (*i.e.*, fusion between liposomes promoted by the magnetic field is limited to a few percent) one may write $r_{0}^{3}\approx a^{2}c$ and hence $a\approx\left( r_{0}^{3}\frac{a}{c} \right)^{\frac{1}{3}}$and $c\approx\left( r_{0}^{3}\frac{c^{2}}{a^{2}} \right)^{\frac{1}{3}}$. The average elasticity modulus $k_{C}$ may then be calculated:

$k_{C}\approx- f\frac{r_{0}^{3}\Delta\chi bB_{0}^{2}}{\mu_{0}({c-a)}}$. In the case of liposomes with *n* lamellae the calculated elasticity modulus translates into $k_{C}^{lip}=nk_{C}$ ^20^.

**Annihilation of magnetic deformation by magic angle sample spinning.**

The deformation of initially spherical liposomes by magnetic field is obtained because the magnetic energy^18^:

*Δχ*: anisotropy of magnetic susceptibility

*b*: bilayer thickness

*B_0_*: magnetic field intensity

*μ_0_*: vacuum permeability

*r_0_*: radius of initial spherical liposomes

*c-a*: difference of ellipsoid long and short axes

$$E_{H}=- \pi\Delta\chi b\frac{B_{0}^{2}}{\mu_{0}}\left[ \left( \frac{2}{3}r_{0}^{2}-\frac{8r_{0}\left( c-a \right)}{5} \right) \right]$$

is slightly greater than the membrane curvature elastic energy:

*r_0_*: radius of initial spherical liposomes

*k_C_*: elastic modulus

*c-a*: difference of ellipsoid long and short axes

*c_0_*: membrane curvature at rest, for symmetric bilayers (*c_0_* = 0) and for asymmetric bilayers (*c_0_* = 2/*r_0_*).

$E_{C}= \pi k_{C}\left[ 2\left( 2-r_{0}c_{0} \right)^{2})+\frac{\frac{9}{4}\left( c-a \right)^{2}}{r_{0}^{2}}\left( \frac{48}{5}-\frac{8\left( r_{0}c_{0} \right)}{5} \right) \right]$

Both energies are computed to be in the range **10^-18^-10^-19^ J** per liposome.

When spinning the sample at a rate of 1.4 kHz and subsequently recording an NMR spectrum, the deformation is cancelled. Let us compute the mechanic energy provided by the spinning speed.

The Mechanical Energy or Kinetic Angular Energy can be written as:

*I* : moment of inertia

*m* : mass of sample (*m=ρV*)

*ρ* : sample density (1030 kg.m^-3^)

*V* = sample volume (100 μL)

$r_{cyl}$: cylindrical NMR rotor inner radius (1.5 mm)

$\nu_{r}$ : spinning speed (1400 Hz

$E_{K}= \frac{1}{2}I\omega^{2}=\frac{1}{4}mr_{cyl}^{2}{(2\pi\nu_{r})}^{2}$

One computes *E_K_* = 4.5 10^-3^ J, which is the kinetic angular energy for N liposomes, *N_MLV_*, inside the sample. Let us now compute the number of liposomes, *N_MLV_*:

$n_{mol}^{sample}$ : total number of lipid molecules in sample

$n_{mol}^{MLV}$ : number of lipids per spherical MLV of area, *S_sphere_*, and composed of ca. *n_b_* ≈ 10-20 concentric bilayers

$$N_{MLV} =n_{mol}^{sample}/n_{mol}^{MLV}$$

$n_{mol}^{sample}$= *N_a_ . m_lipids_/mw_lipid_* = (10 10^-3^ g/700 g.mole^-1^) x 6.02 10^23^ molecules.mole^-1^ = 8.6 10^18^ molecules. By considering that each lipid in the membrane occupies a surface of 60 Å^2^, one obtains:

$n_{mol}^{MLV}$= 2*n_b_*.*S_sphere_/A_lipid_* = 40 x 4π$r_{0}^{2}$/*A_lipid_* =40 x 4π (0.45 10^−6^ μ)^2^/60 Å^2^ = 4.2 10^7^ molecules, and

$N_{MLV}$ ≈ 2 10^11^

The energy per liposome due to MAS at 1.4 kHz is then: *E_K_*/$N_{MLV}$ ≈ **2 10^-14^ J**, a value much higher than both magnetic and curvature elastic energies (*vide supra*).

References

1 Kaffarnik, S., Ehlers, I., Grobner, G., Schleucher, J. & Vetter, W. Two-Dimensional P-31,H-1 NMR Spectroscopic Profiling of Phospholipids in Cheese and Fish. *J. Agric. Food Chem.* **61**, 7061-7069, doi:10.1021/jf4021812 (2013).

2 Meneses, P. & Glonek, T. High-Resolution P-31 NMR of Extracted Phospholipids. *J. Lipid Res.* **29**, 679-689 (1988).

3 Massiot, D. *et al.* Modelling one- and two-dimensional solid-state NMR spectra. *Magnetic Resonance in Chemistry* **40**, 70-76, doi:10.1002/mrc.984 (2002).

4 Casu, M., Anderson, G. J., Choi, G. & Gibbons, W. A. Nmr Lipid Profiles of Cells, Tissues and Body-Fluids .1. 1D and 2dD Proton NMR of Lipids from Rat-Liver. *Magnetic Resonance in Chemistry* **29**, 594-602, doi:10.1002/mrc.1260290610 (1991).

5 Edzes, H. T., Teerlink, T. & Valk, J. Phospholipid Identification in Tissue-Extracts by 2-Dimensional P-31-H-1 NMR-Spectroscopy with Isotropic Proton Mixing. *Journal of Magnetic Resonance* **95**, 387-395, doi:10.1016/0022-2364(91)90228-l (1991).

6 [*https://sdbs.db.aist.go.jp*](https://sdbs.db.aist.go.jp) *(National Institute of Advanced Industrial Science and Technology,date of access)*.

7 Douliez, J. P., Leonard, A. & Dufourc, E. J. Restatement of Order Parameters in Biomembranes - Calculation of C-C Bond Order Parameters from C-D Quadrupolar Splittings. *Biophys. J.* **68**, 1727-1739, doi:10.1016/s0006-3495(95)80350-4 (1995).

8 Douliez, J. P., Leonard, A. & Dufourc, E. J. Conformational order of DMPC sn-1 versus sn-2 chains and membrane thickness: An approach to molecular protrusion by solid state H-2-NMR and neutron diffraction. *J. Phys. Chem.* **100**, 18450-18457, doi:10.1021/jp961220v (1996).

9 Davis, J. H. The Description of Membrane Lipid Conformation, Order and Dynamics by 2H-NMR. *Biochimica Et Biophysica Acta* **737**, 117-171, doi:10.1016/0304-4157(83)90015-1 (1983).

10 Burnett, L. J. & Muller, B. H. Deuteron Quadrupole Coupling Constants in 3 Solid Deuterated Paraffin Hydrocarbons-C2D6, C4D10, C6D14. *J. Chem. Phys.* **55**, 5829-&, doi:10.1063/1.1675758 (1971).

11 Douliez, J. P., Leonard, A. & Dufourc, E. J. Restatement of order parameters in biomembranes: calculation of C-C bond order parameters from C-D quadrupolar splittings. *Biophysical Journal* **68**, 1727-1739 (1995).

12 Shin, K. H., Fujiwara, T. & Akutsu, H. Modulation of the Specific Interaction of Cardiolipin with Cytochrome-C by Zwitterionic Phospholipids in Binary Mixed Bilayers - A 2-H and P-31 NMR-Study. *Journal of Molecular Structure* **355**, 47-53, doi:10.1016/0022-2860(95)08866-t (1995).

13 Tilcock, C. P. S. & Cullis, P. R. The Polymorphic Phase-Behavior of Mixed Phosphatidylserine-Phosphatidylethanolamine Model Systems as Detected by P-31-NMR - Effects of Divalent-Cations and pH. *Biochimica Et Biophysica Acta* **641**, 189-201, doi:10.1016/0005-2736(81)90583-6 (1981).

14 Cullis, P. R. & Dekruyff, B. P-31 NMR-Studies of Unsonicated Aqueous Dispersions of Neutral And Acidic Phospholipids - Effects of Phase-Transitions, P2H and Divalent-Cations on Motion In Phosphate Region of Polar Headgroup. *Biochimica Et Biophysica Acta* **436**, 523-540, doi:10.1016/0005-2736(76)90438-7 (1976).

15 Zhendre, V. *et al.* Key Role of Polyphosphoinositides in Dynamics of Fusogenic Nuclear Membrane Vesicles. *PLoS One* **6**, doi:10.1371/journal.pone.0023859 (2011).

16 Pott, T. & Dufourc, E. J. Action of Melittin on the DPPC-Cholesterol Liquid-Ordered Phase - A Solid-State 2H-NMR and P-31-NMR Study. *Biophys. J.* **68**, 965-977, doi:10.1016/s0006-3495(95)80272-9 (1995).

17 Vist, M. R. & Davis, J. H. Phase-Equilibria of Cholesterol Dipalmitoylphosphatidylcholine Mixtures - 2H Nuclear Magnetic-Resonance and Differential Scanning Calorimetry. *Biochemistry* **29**, 451-464, doi:10.1021/bi00454a021 (1990).

18 Helfrich, W. Lipid Bilayer Spheres - Deformation and Birefringence in Magnetic-Fields. *Phys. Lett. A* **A 43**, 409-410, doi:10.1016/0375-9601(73)90396-4 (1973).

19 Helfrich, W. Elastic Properties of Lipid Bilayers - Theory and Possible Experiments. *Zeitschrift Fur Naturforschung C-a Journal of Biosciences* **C 28**, 693-703 (1973).

20 Boroske, E. & Helfrich, W. Magnetic-Anisotropy of Egg Lecithin Membranes. *Biophys. J.* **24**, 863-868, doi:10.1016/s0006-3495(78)85425-3 (1978).
